# Sesamin Ameliorates High‐Sugar, High‐Fat Diet‐Induced Hepatic Dysfunction via CYP1A2‐Mediated Regulation of Lipid Metabolism and Oxidative Stress

**DOI:** 10.1111/jcmm.71274

**Published:** 2026-07-17

**Authors:** Xiliang Zhu, Jie Yan, Qi Liu, Zhaoyun Cheng, Yi Luo

**Affiliations:** ^1^ Department of Cardiovascular Surgery, Heart Center of Henan Provincial People's Hospital, Fuwai Central China Cardiovascular Hospital Central China Fuwai Hospital of Zhengzhou University Zhengzhou China; ^2^ Department of Clinical Nutrition, Luzhou People's Hospital Luzhou China; ^3^ Department of Cardiology, Sir Run Shaw Hospital, School of Medicine Zhejiang University Hangzhou China; ^4^ Department of Cardiology, the Affiliated Hospital, Key Laboratory of Medical Electrophysiology of the Ministry of Education, Medical Electrophysiological Key Laboratory of Sichuan Province, Institute of Cardiovascular Research Southwest Medical University Luzhou China

**Keywords:** CYP1A2, lipid, proteome, sesamin

## Abstract

BackgroundHigh‐sugar, high‐fat diets (HSDFD) can lead to hepatic dysfunction, but the molecular mechanisms underlying protective compounds like sesamin remain unclear MethodsWe employed a multi‐faceted approach combining in silico and in vivo techniques. Utilizing the AlphaFold database, we screened multi‐species proteomes to identify potential sesamin targets. Molecular dynamics simulations and MM‐PBSA analysis were used to characterize sesamin‐protein interactions. In vivo studies included single‐cell RNA sequencing of hepatocytes from HSDFD‐fed mice. We validated our findings using cellular thermal shift assays (CETSA), drug affinity responsive target stability (DARTS) assays, Western blotting, and qPCR. ResultsHSDFD induced significant redistribution of hepatocyte subpopulations with high CYP1A2 abundance. Computational analysis identified CYP1A2 as a direct target of sesamin, with species‐specific interaction patterns. Sesamin exhibited stable binding to CYP1A2, modulating its activity through subtle conformational changes. Key residues F451 and I386 were crucial for sesamin's regulation of CYP1A2‐mediated ROS production. Sesamin supplementation ameliorated HSDFD‐induced hepatic dysfunction by modulating fatty acid metabolism and oxidative stress responses. ConclusionOur study unveils CYP1A2 as a key molecular target of sesamin in HSDFD‐induced hepatic dysfunction, providing new insights into potential therapeutic strategies for metabolic liver disorders.

## 1 Introduction

Sesamin, a principal lignan found in sesame seeds ( *Sesamum indicum* L.), has attracted considerable attention due to its diverse biological activities and potential health benefits [1, 2, 3, 4, 5, 6]. This natural compound, first isolated in the 1950s, is characterized by its unique structure comprising two methylenedioxyphenyl groups linked by a furofuran skeleton [7, 8]. While primarily present in sesame seeds, sesamin is also found in smaller quantities in other plant sources such as pumpkin seeds and flaxseeds.

Extensive research has highlighted sesamin's promising effects in combating various metabolic disorders [2, 5, 6, 9, 10]. Studies have demonstrated its potential in improving lipid profiles, reducing inflammation, and exhibiting antioxidant properties [9, 11, 12, 13, 14, 15]. These findings position sesamin as a compound of interest in the prevention and management of metabolic syndrome, a cluster of conditions including obesity, hypertension, dyslipidemia, and insulin resistance [5, 6, 11, 12, 16]. Additionally, sesamin has shown promise in mitigating liver steatosis, regulating glucose metabolism, and even possessing anti‐cancer properties in certain models.

Despite the growing evidence supporting sesamin's beneficial effects on metabolic health, the precise mechanisms underlying its action remain largely unclear. While some studies have suggested interactions with specific proteins or pathways, such as peroxisome proliferator‐activated receptors (PPARs) or antioxidant enzymes, a comprehensive understanding of sesamin's molecular targets across different species is lacking [17, 18, 19]. This knowledge gap hinders the full exploitation of sesamin's therapeutic potential and limits our ability to predict its efficacy and safety across different model organisms and in humans.

Recent advancements in computational biology, particularly in protein structure prediction, have opened new avenues for investigating small molecule‐protein interactions on a proteome‐wide scale. AlphaFold, a deep learning‐based approach developed by DeepMind, has revolutionized structural biology by achieving unprecedented accuracy in predicting protein structures [20, 21]. The public release of the AlphaFold Protein Structure Database, containing high‐confidence structural predictions for numerous species, provides an invaluable resource for researchers across various disciplines, including drug discovery and comparative biology [21, 22].

This study conducts comprehensive virtual screening of sesamin against entire proteomes of mice, rats, zebrafish, and humans using AlphaFold‐predicted structures. Our objectives are to identify novel protein targets explaining sesamin's metabolic effects, investigate species‐specific interactions, and validate computational predictions experimentally. By combining AI‐driven structural biology with pharmacological approaches, we aim to elucidate sesamin's molecular mechanisms, potentially guiding future drug design for metabolic disorders and establishing a new paradigm for natural product research.

## 2 Methods

### 2.1 Animal Model and Dietary Intervention

All animal experiments were approved by the Animal Ethics Committee of the Second Affiliated Hospital, Zhejiang University School of Medicine, and were performed in accordance with institutional guidelines and the ARRIVE recommendations.

Male C57BL/6 mice (8 weeks old; SLAC, Shanghai, China) were housed under specific pathogen‐free conditions under a 12 h light/12 h dark cycle at controlled temperature and humidity, with free access to food and water. After acclimatization, mice were randomly assigned to four groups: (1) Chow, (2) Chow+SES, (3) HSDFD, and (4) HSDFD+SES.

The HSDFD used in this study was based on the Research Diets D12079B Western diet (Research Diets, New Brunswick, NJ, USA). According to the manufacturer's formulation, this diet contains 21% fat by weight (200 g/kg anhydrous butter fat plus 10 g/kg corn oil), 35% sucrose by weight (350 g/kg), and 1.5 g/kg added cholesterol, corresponding to an overall cholesterol content of approximately 0.21%; the caloric distribution is 17% kcal from protein, 40% kcal from fat, and 43% kcal from carbohydrate, with an energy density of 4.67 kcal/g. Notably, this formulation does not contain sodium cholate. The control group received standard chow.

Sesamin (SES; S25758, Yuanye, Shanghai, China) was administered orally through diet supplementation at a concentration of 10 g/kg diet throughout the intervention period. Thus, mice in the Chow+SES and HSDFD+SES groups received SES continuously for 6 months, whereas mice in the Chow and HSDFD groups received the corresponding diets without SES. Body weight was recorded weekly throughout the study.

For endpoint analyses, mice were euthanized after 6 months of dietary intervention under isoflurane anaesthesia followed by cervical dislocation, and liver tissues were harvested for histopathological and biochemical analyses. In addition, for time‐course molecular analyses, liver tissues were collected at 0, 2, 4, and 6 months during HSDFD feeding for western blot detection of CYP1A2‐, oxidative stress response‐, and lipid metabolism‐related proteins. Histopathological analyses shown in the main comparison were performed using liver tissues collected at the 6‐month endpoint.

Serum samples were collected at sacrifice for lipid profiling and liver function‐related measurements. Portions of liver tissue were fixed in 4% paraformaldehyde for histology, while the remaining tissues were snap‐frozen in liquid nitrogen and stored at −80°C for subsequent molecular analyses.

### 2.2 In Vitro Experimentation

Mouse hepatocyte AML12 cells were cultured in DMEM/F‐12 medium supplemented with 10% fetal bovine serum (FBS), 1× insulin‐transferrin‐selenium (ITS), 40 ng/mL dexamethasone, and 1% penicillin/streptomycin at 37°C in a humidified incubator with 5% CO_2_. Human hepatocyte THLE‐2 cells were maintained in collagen I‐coated culture dishes using the recommended complete medium under the same culture conditions. Primary mouse hepatocytes were isolated from male C57BL/6 mice using a standard two‐step collagenase perfusion method. Briefly, livers were perfused in situ with prewarmed calcium‐free buffer followed by collagenase‐containing digestion buffer, and the dissociated cells were filtered and collected by low‐speed centrifugation. Viable hepatocytes were seeded onto collagen‐coated plates and cultured in Williams' E medium supplemented with 10% FBS, 1× ITS, dexamethasone, and 1% penicillin/streptomycin. Stable AML12 cell lines with Cyp1a2 overexpression or Cyp1a2 knockout were generated by Haixing Biotechnology. The overexpression cell line was established by stable transduction with a vector containing the full‐length mouse Cyp1a2 coding sequence, whereas the knockout cell line was generated using a CRISPR/Cas9‐based gene‐editing strategy targeting mouse Cyp1a2. The efficiency of Cyp1a2 overexpression and knockout was confirmed by western blotting before subsequent experiments. For lipid overload experiments, AML12 cells, THLE‐2 cells, or primary mouse hepatocytes were treated with oleic acid (OA; S31608, Yuanye, Shanghai, China) conjugated to fatty acid‐free bovine serum albumin (BSA) at the indicated concentrations and durations. Sesamin (SES; S25758, Yuanye, Shanghai, China) was added at the indicated concentrations (100 nM–10 μM) according to the experimental design. In selected experiments, additional treatments, including H_2_O_2_, N‐acetyl‐L‐cysteine (NAC), or CYP1A2‐related compounds, were applied as specified in the corresponding figure legends. Control cells received the corresponding vehicle treatment.

### 2.3 Computational Screening and Analysis

Complete proteome structures for mouse, rat, zebrafish, and human were obtained from the AlphaFold database. Potential binding pockets were identified using fpocket, and AutoDock Vina was used for virtual screening of SES against these structures.

### 2.4 Hepatic Histopathological Evaluation

Liver tissues were processed for Oil Red O, H&E, Masson's trichrome, and Sirius Red staining. Quantitative analysis was performed using ImageJ software. Statistical analysis involved one‐way ANOVA with Tukey's post hoc test (*p* < 0.05).

### 2.5 CYP1A2 Immunohistochemistry and Immunofluorescence

Liver sections were stained with CYP1A2 antibody (Cat No. 19936–1‐AP, Proteintech, Wuhan, China) and HRP‐conjugated secondary antibody (Cat No. RGAR001, Wuhan, China). DAB substrate (P0202, Beyotime, Shanghai, China) was used for visualization. For immunofluorescence, AML12 cells were stained with CYP1A2 antibody and CoraLite@Plus 488‐conjugated secondary antibody (Cat No. RGAR002, Proteintech, Wuhan, China). Images were captured using a confocal microscope and analysed with ImageJ.

### 2.6 Biochemical Analyses

Serum levels of ALB, ALP, ALT, AST, TP, HDL‐C, LDL‐C, TC, and TG were measured. Liver tissue samples were analysed for TC, TG, SOD activity, GPx activity, MDA levels, and T‐AOC using commercial kits (Jiancheng, Nanjing, China). Protein concentrations were determined by Bradford assay.

### 2.7 Cellular Lipid Accumulation Assays

Lipid accumulation in AML12 cells was assessed using Oil Red O staining (C0157S, Beyotime, Shanghai, China) and BODIPY staining (C2053S, Beyotime, Shanghai, China). Oil Red O stained cells were quantified spectrophotometrically at 510 nm. BODIPY‐stained cells were analysed by confocal microscopy and flow cytometry. ImageJ and FlowJo software were used for image and flow cytometry data analysis, respectively.

### 2.8 Cellular Thermal Shift Assay (CETSA)

CETSA assessed SES's effect on CYP1A2 thermal stability in AML12 cells. Cells treated with SES were exposed to a temperature gradient (37°C–70°C). Soluble protein fractions underwent Western blot analysis for CYP1A2. Thermal stability curves were plotted to derive melting temperatures (Tm), indicating potential SES‐CYP1A2 interactions.

### 2.9 Drug Affinity Responsive Target Stability (DARTS) Assay

DARTS investigated direct SES‐CYP1A2 interactions. AML12 cell lysates were incubated with SES or vehicle, followed by limited proteolysis with pronase (S10014, Yuanye, Shanghai, China). CYP1A2 levels were analysed by Western blotting to assess SES‐induced protection against proteolysis.

### 2.10 Overexpression of CYP1A2 Variants

Plasmids expressing wild‐type human CYP1A2 and mutants CYP1A2 (I386A) and CYP1A2 (F451A) were constructed. AML12 cells were transfected and confirmed for expression. Transfected cells underwent SES treatment followed by functional assays. Data were analysed using one‐way ANOVA with Tukey's post hoc test (*p* < 0.05).

### 2.11 Gene Expression Analysis by Quantitative PCR

RNA from AML12 cells was extracted using TRIzol and reverse‐transcribed. QPCR using SYBR Green master mix analysed expression of XDH, CYP1A1, CYP1A2, CYBA, PTGS2, MAOA, and MAOB. Four conditions were tested: vehicle control (VEH), sesamin (VEH + SES), oleic acid (OA), and OA + SES. Gene expression was normalized and calculated using the 2^(‐ΔΔCt) method. Data were analysed by one‐way ANOVA with Tukey's post hoc test (significance: * *p* < 0.05, ** *p* < 0.01, *** *p* < 0.001).

### 2.12 Western Blotting Analysis of Protein Expression

Protein expression was analysed by western blotting in liver tissues or cultured hepatocytes under the indicated experimental conditions. Total protein was extracted using RIPA lysis buffer supplemented with protease and phosphatase inhibitors. Protein concentrations were determined using a BCA protein assay kit. Equal amounts of protein were separated by SDS‐PAGE and transferred onto PVDF membranes. After blocking with 5% non‐fat milk or bovine serum albumin in TBST, membranes were incubated overnight at 4°C with the following primary antibodies: CYP1A2 (Cat No. 19936–1‐AP, Proteintech), GAPDH (Cat No. 60004–1‐Ig, Proteintech), NRF2 (AF0639, Affinity), GPX4 (DF6701, Affinity), NQO1 (DF6437, Affinity), SOD1 (Cat No. 10269–1‐AP, Proteintech), SOD2 (Cat No. 24127–1‐AP, Proteintech), FABP1 (Cat No. 13626–1‐AP, Proteintech), SCD1 (Cat No. 28678–1‐AP, Proteintech), PGC1α (Cat No. 66369–1‐Ig, Proteintech), and PPARα (AF5301, Affinity).

After washing with TBST, membranes were incubated with the appropriate HRP‐conjugated secondary antibodies at room temperature. Immunoreactive bands were detected using an enhanced chemiluminescence (ECL) system and quantified using ImageJ software. Target protein expression was normalized to GAPDH as the internal loading control.

Western blot experiments were repeated independently at least three times. Statistical analyses are described in the corresponding figure legends.

### 2.13 Single‐Cell Transcriptomics Analysis

We analysed scRNA‐seq data (GEO: GSE182365) comparing liver cells from mice fed standard chow versus high‐fat, high‐sucrose diets. After preprocessing and quality control, we constructed a liver cell atlas using UMAP for dimensionality reduction. Differential expression analysis identified diet‐induced changes in hepatic cell populations, focusing on metabolic pathways and transcriptional networks associated with liver disease.

### 2.14 Molecular Dynamics Simulation

Molecular dynamics simulations of CYP1A2‐sesamin interactions were performed using GROMACS. The CYP1A2 structure (PDB ID: 2HI4) was simulated with and without sesamin for 100 ns. We analysed RMSD, RMSF, SASA, hydrogen bonds, binding pocket dynamics, and Gibbs free energy landscapes to evaluate structural stability and conformational changes. Comparative analysis between apo‐CYP1A2 and the CYP1A2‐sesamin complex elucidated sesamin‐induced alterations in CYP1A2 dynamics.

## 3 Result

### 3.1 Transcriptomic Profiling Reveals Sesamin's Impact on Hepatic Lipid Metabolism

**FIGURE 1:**
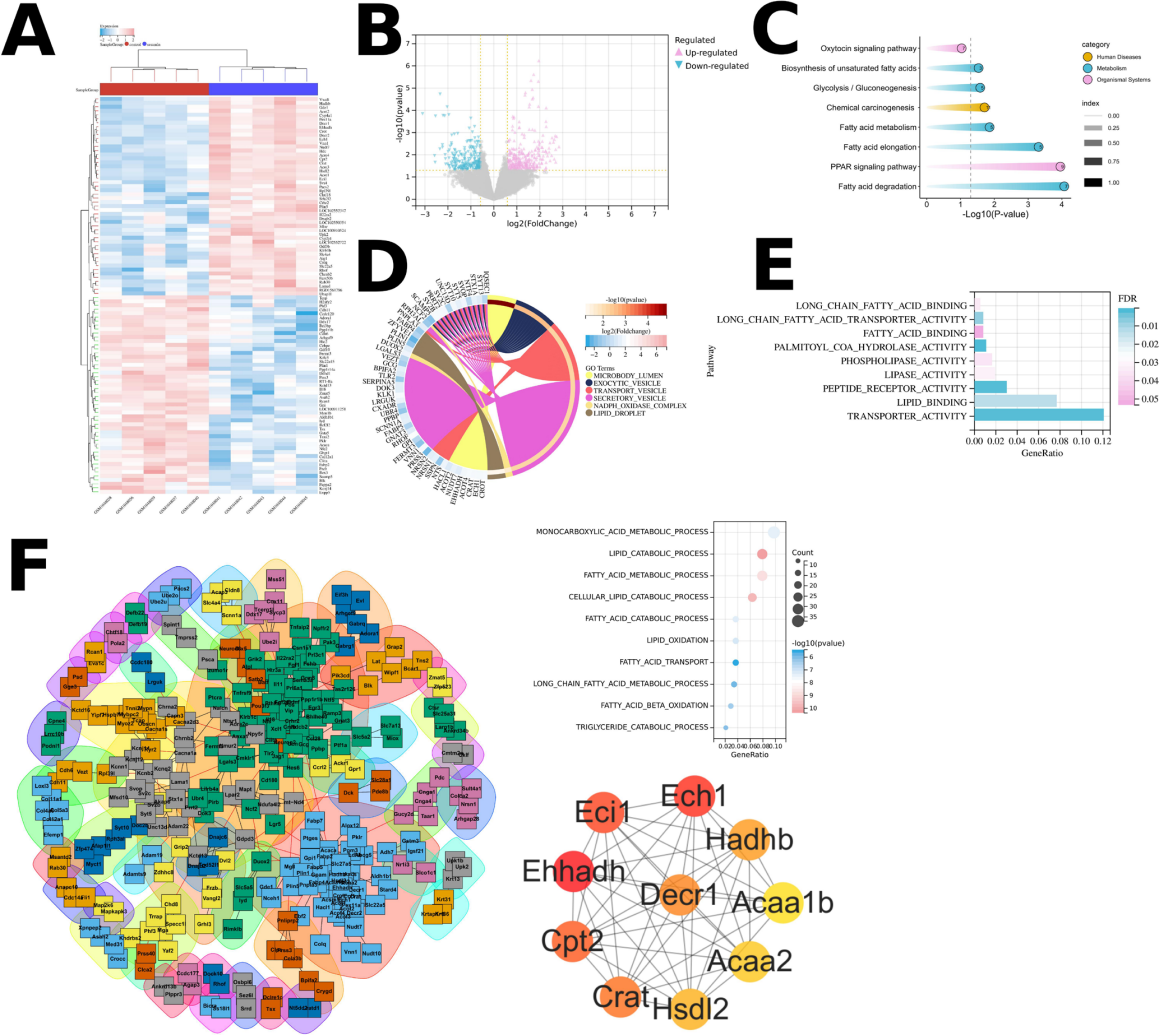
Transcriptomic analysis of mouse liver under different treatment conditions. (A) Heatmap showing differential gene expression in mouse liver across different treatment groups. Red indicates upregulation, blue indicates downregulation. (B) Volcano plot of differentially expressed genes in mouse liver. Red dots represent upregulated genes, blue dots represent downregulated genes. (C) KEGG pathway enrichment analysis of differentially expressed genes in mouse liver. Bar plot shows significantly enriched pathways, with colour indicating different categories and bar length representing statistical significance. (D) Gene Ontology (GO) cellular component (CC) enrichment analysis depicted as a circular plot. Different colours represent various cellular components enriched in differentially expressed genes. (E) GO molecular function (MF) analysis of terms enriched in differentially expressed genes in mouse liver. Bar plot shows enriched GO terms related to lipid metabolism and transport. (F) Gene network and pathway analysis in mouse liver. Left: Network visualization of interacting genes affected by treatment conditions. Right upper: Bubble plot showing enriched biological processes (BP) related to lipid metabolism, including fatty acid β‐oxidation. The size of the bubbles indicates the gene count, while the colour represents the statistical significance. Right lower: Hub gene network forming a fatty acid β‐oxidation cluster, highlighting key genes involved in this process.

Transcriptomic analysis of mouse liver tissue revealed significant alterations in gene expression patterns across different treatment groups, including sesamin supplementation (Figure 1A,B). The heatmap and volcano plot visualizations demonstrated a wide range of both up‐ and down‐regulated genes, indicating a broad impact of sesamin on hepatic gene expression profiles.

KEGG pathway enrichment analysis highlighted several lipid metabolism‐related pathways significantly affected by sesamin treatment (Figure 1C). These included fatty acid metabolism, fatty acid elongation, PPAR signalling pathway, and fatty acid degradation. The enrichment of these pathways suggests that sesamin exerts a substantial modulatory effect on genes involved in lipid metabolism within the liver.

Gene Ontology (GO) analysis of cellular components and molecular functions further corroborated sesamin's impact on lipid metabolism (Figure 1D,E). Enriched terms included long‐chain fatty acid binding, fatty acid transporter activity, and lipase activity. These findings indicate that sesamin influences the expression of genes involved in fatty acid transport, binding, and breakdown processes.

Gene network analysis revealed complex interactions among differentially expressed genes, underscoring the widespread effects of sesamin on liver gene expression (Figure 1F, left). Notably, the bubble plot and hub gene network analysis highlighted a significant enrichment in fatty acid β‐oxidation processes (Figure 1F, right). Key genes in this network included Acaa1b, Acaa2 (acetyl‐CoA acyltransferases), Hadha, Hadhb (hydroxyacyl‐CoA dehydrogenases), and Cpt2 (carnitine palmitoyltransferase 2). The upregulation of these genes, crucial for fatty acid breakdown, suggests that sesamin may enhance fatty acid oxidation in the liver.

### 3.2 Cross‐Species Analysis Reveals Conserved High‐Affinity Interaction Between Sesamin and CYP1A2

**FIGURE 2:**
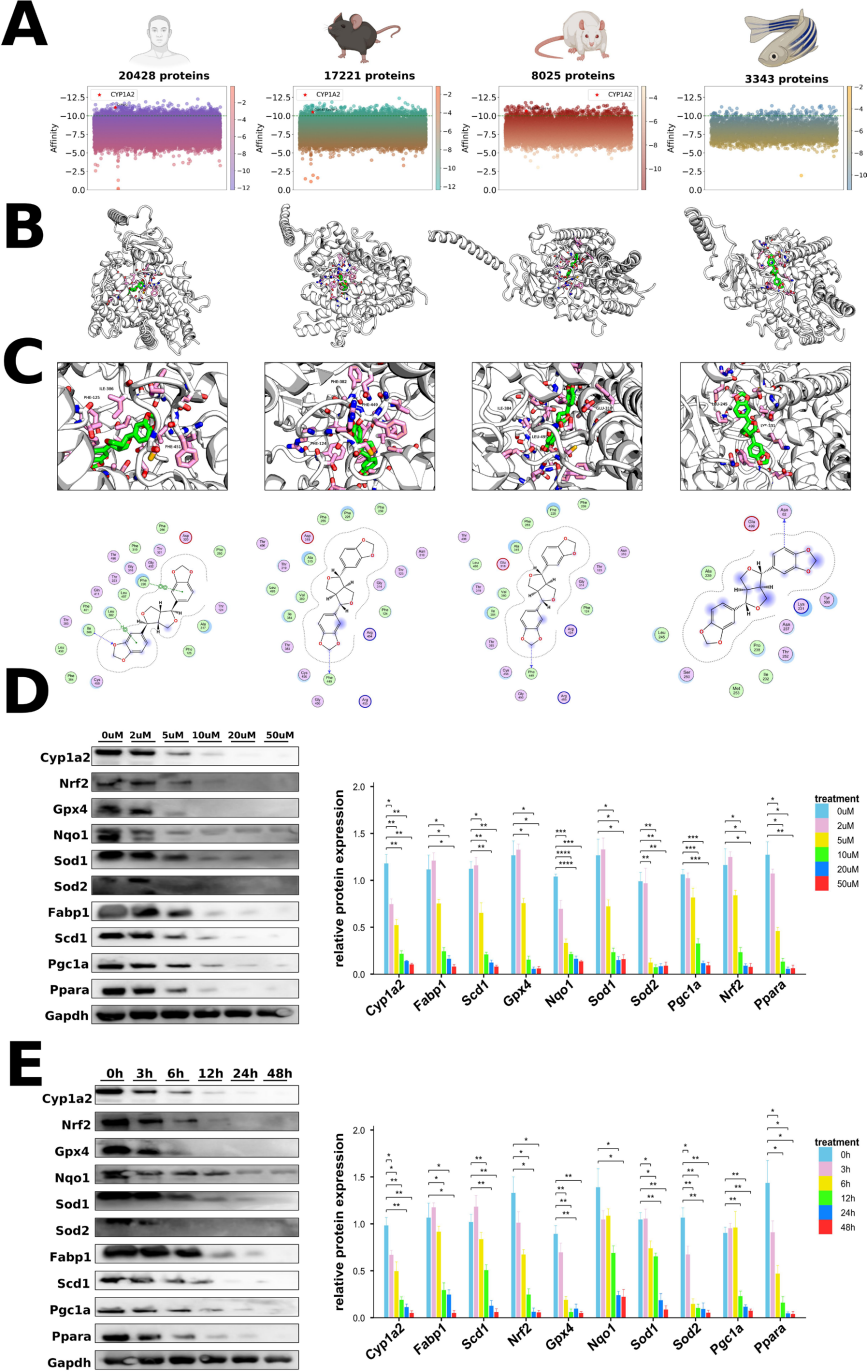
Cross‐species analysis of sesamin–CYP1A2 interactions and validation in oleic acid‐treated primary mouse hepatocytes. (A) Docking affinity distribution of sesamin against the proteomes of human, mouse, rat, and zebrafish. CYP1A2 (red) exhibits high binding affinity in human, mouse, and rat, but not in zebrafish. (B) Three‐dimensional views of CYP1A2 binding pockets (grey) with docked sesamin (green) across species. (C) Detailed sesamin–CYP1A2 interaction patterns. Upper: 3D views of the binding interface and key interacting residues. Lower: 2D interaction maps showing residue–ligand contacts. (D) Dose‐dependent effects of sesamin on CYP1A2 and proteins related to oxidative stress response and lipid metabolism in an oleic acid‐induced lipid overload model of primary mouse hepatocytes. Representative Western blots are shown on the left, and quantitative analyses are shown on the right. (E) Time‐course effects of sesamin (10 μM) on CYP1A2 and proteins related to oxidative stress response and lipid metabolism in oleic acid‐treated primary mouse hepatocytes. Representative Western blots are shown on the left, and quantitative analyses are shown on the right.

Proteome‐wide virtual screening across human, mouse, rat, and zebrafish revealed that sesamin showed high predicted binding affinity for CYP1A2 in human, mouse, and rat, whereas no comparable high‐affinity interaction was identified for the zebrafish homologue CYP1A (Figure 2A). These results suggest that CYP1A2 may represent a conserved mammalian target of sesamin.

Structural analysis further demonstrated that sesamin occupied a comparable binding cavity in mammalian CYP1A2 proteins, although subtle species‐specific differences were observed (Figure 2B,C). In human CYP1A2, sesamin was positioned within a semi‐enclosed pocket that was partially solvent exposed. The ligand was stabilized by π–H interactions with Phe226 and Leu362 and a hydrogen bond with Ile386, together with extensive hydrophobic and polar contacts within the surrounding pocket (Figure 2C).

In mouse and rat CYP1A2, sesamin adopted highly similar binding conformations. In both species, sesamin was located in a semi‐enclosed cavity and formed a key hydrogen bond interaction with Phe449, which corresponds to Phe451 in human CYP1A2 based on sequence alignment. The surrounding residues also created a conserved hydrophobic‐polar microenvironment, supporting a highly similar binding mode in the two rodent orthologs (Figure 2C).

In contrast, zebrafish CYP1A, which lacks a direct CYP1A2 ortholog, exhibited a distinct and weaker predicted interaction pattern. The ligand‐binding region appeared more discontinuous, with the pocket divided into two parts, and sesamin formed only a limited interaction, including a hydrogen bond with Asn62 (Figure 2C). These data indicate that the sesamin–CYP1A2 interaction is evolutionarily conserved in mammals but less well‐preserved in zebrafish.

To further validate the biological relevance of CYP1A2 in a lipid‐overload context, we examined the effects of sesamin in oleic acid (OA)‐treated primary mouse hepatocytes. Sesamin increased CYP1A2 expression in a dose‐dependent manner and concomitantly modulated proteins involved in oxidative stress response and lipid metabolism (Figure 2D). Time‐course analysis further showed that 10 μM sesamin dynamically regulated CYP1A2 and these downstream protein changes in OA‐treated primary mouse hepatocytes (Figure 2E). Together, these findings support a conserved sesamin–CYP1A2 interaction across mammalian species and suggest that CYP1A2 is associated with the regulation of oxidative stress and lipid metabolic homeostasis under lipid overload conditions.

### 3.3 Single‐Cell RNA Sequencing Reveals HSDFD‐Induced Transcriptional Remodelling of Hepatocyte Subpopulations

**FIGURE 3:**
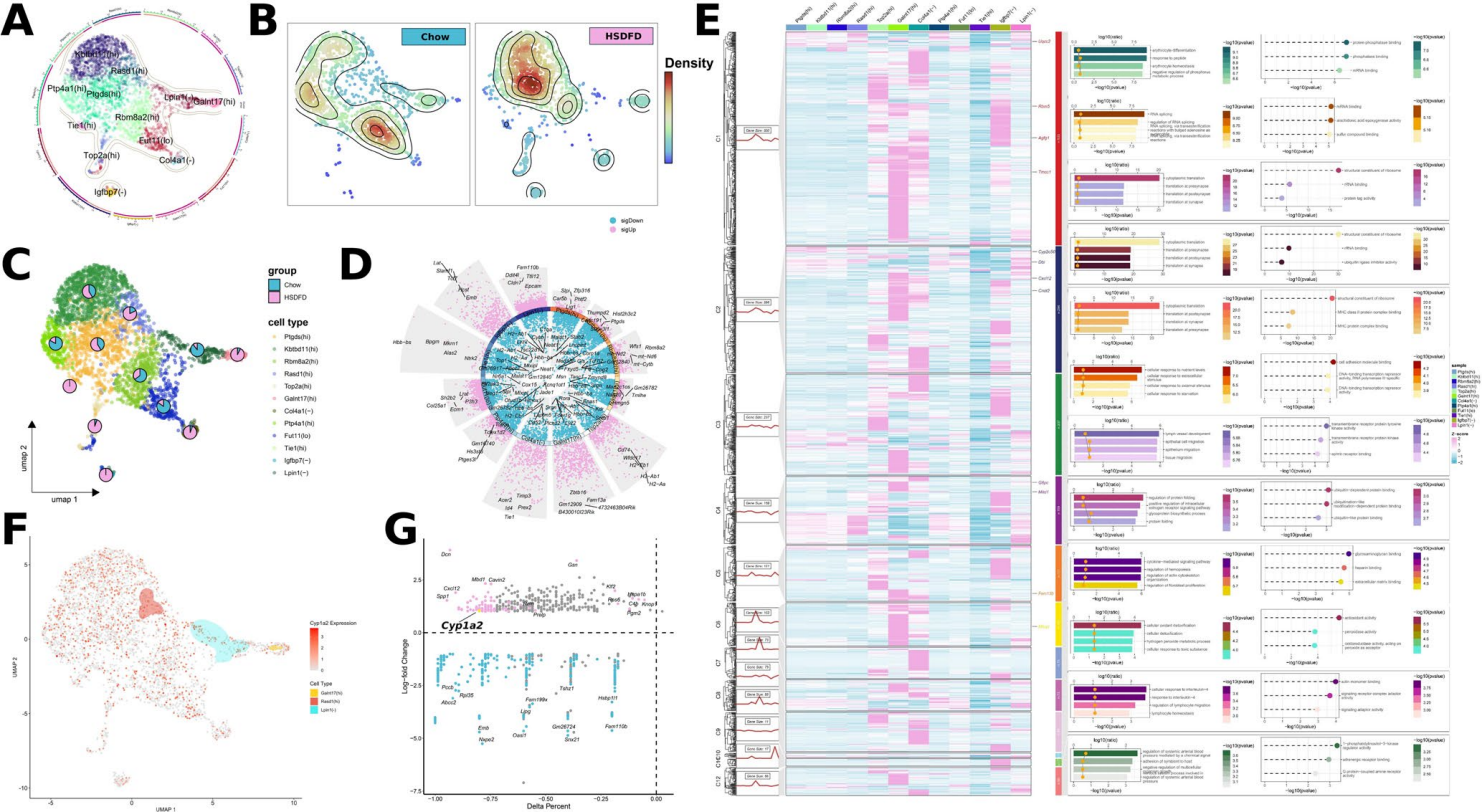
Single‐cell RNA sequencing analysis of mouse liver under chow and high‐sugar, high‐fat diet (HSHFD) conditions. (A) Circular plot showing hepatocyte subpopulation distribution in the liver. (B) Density plots illustrating changes in subpopulation cell density between chow and HSHFD conditions. (C) UMAP plot depicting shifts in hepatocyte subpopulation proportions between chow and HSHFD groups. (D) Circular heatmap showing marker gene expression across different hepatocyte subpopulations under chow and HSHFD conditions. (E) Heatmap and bar plots illustrating biological process (BP) and molecular function (MF) enrichment of marker genes in different hepatocyte subpopulations under chow and HSHFD conditions. (F) UMAP plot showing CYP1A2 expression across hepatocyte subpopulations. (G) Scatter plots demonstrating significant upregulation of CYP1A2 in the Igfbp7 (−) subpopulation after HSHFD induction.

Single‐cell RNA sequencing analysis of mouse liver revealed significant transcriptional remodelling of hepatocyte subpopulations in response to a high‐sugar, high‐fat diet (HSDFD) (Figure 3). Our analysis identified multiple heterogeneous subpopulations of hepatocytes within the liver under both chow and HSDFD conditions (Figure 3A).

HSDFD induction led to marked changes in the density distribution of hepatocyte subpopulations, resulting in the emergence of several isolated single‐cell subpopulations, including Col4a1 (−), Igfbp7 (−), and Galnt17 (hi) clusters (Figure 3B). This shift in subpopulation structure indicates that HSDFD exacerbates hepatocyte heterogeneity in the liver. Moreover, even pre‐existing hepatocyte subpopulations exhibited significant changes in their proportions following HSDFD induction (Figure 3C).

We identified marker genes that were either upregulated or downregulated in different hepatocyte subpopulations after HSDFD treatment (Figure 3D). Gene Ontology enrichment analysis of these marker genes revealed distinct biological processes (BP) and molecular functions (MF) associated with each subpopulation (Figure 3E). Notably, several subpopulations showed enrichment in antioxidant activity and superoxide dismutase activity, while others were enriched in responses to nutrient levels.

CYP1A2, which exhibits high affinity for sesamin, was found to be highly expressed across all hepatocyte subpopulations (Figure 3F). Interestingly, the Igfbp7 (−) subpopulation showed significant upregulation of CYP1A2 expression following HSDFD induction (Figure 3G).

These findings demonstrate that HSDFD induces substantial transcriptional remodelling of hepatocyte subpopulations at the single‐cell level, enhancing cellular heterogeneity and altering the expression of key metabolic and stress‐response genes. The widespread expression of CYP1A2 across hepatocyte subpopulations, coupled with its upregulation in specific subsets after HSDFD treatment, suggests a potential mechanism for the interaction between dietary factors, sesamin, and hepatic metabolism.

### 3.4 Single‐Cell Analysis Reveals Diet‐Induced Metabolic Reprogramming of Hepatocyte Subpopulations

Single‐cell analysis uncovered distinct metabolic profiles and population shifts in hepatocyte subpopulations under chow and high‐sugar, high‐fat diet (HSHFD) conditions. Rasd1 (hi), Galnt17 (hi), and Lipin1 (−) subpopulations exhibited high overall metabolic activity, with Galnt17 (hi) and Lipin1 (−) emerging as novel HSHFD‐induced subsets (Figure S1A). Pathway‐specific analysis revealed that most subpopulations maintained high activity across multiple metabolic pathways, while the newly formed Igfbp7 (−) subpopulation showed suppressed activity specifically in glycolysis, oxidative phosphorylation, and TCA cycle pathways (Figure S1B).

Investigation of fatty acid metabolism pathways demonstrated that the HSHFD‐induced Galnt17 (hi) subpopulation exhibited elevated fatty acid elongation activity (Figure S1C). While fatty acid biosynthesis remained relatively stable (Figure S1D), HSHFD generally intensified fatty acid degradation pathway activity across hepatocyte subsets (Figure S1E). Additionally, HSHFD enhanced cytochrome P450‐mediated xenobiotic metabolism in all subpopulations (Figure S1F).

Mapping mouse hepatocyte subpopulations to the Human Protein Atlas liver cell atlas revealed correspondence to human hepatocytes‐1, ‐2, and ‐3 subtypes (Figure S1G). HSHFD induced a shift towards regions associated with high fatty acid metabolic activity, mirroring human hepatocytes‐1 and ‐2 (Figure S1H, Figure S2). Galnt17 (hi) and Lipin1 (−) subsets primarily drove migration towards hepatocytes‐1, while Igfbp7 (−) shifted towards hepatocytes‐2 (Figure S1I).

These findings highlight the dynamic metabolic reprogramming of hepatocyte subpopulations in response to HSHFD, characterized by novel subsets with distinct metabolic profiles and a shift towards fatty acid metabolism‐associated phenotypes, providing insights into diet‐induced hepatic metabolic adaptations.

### 3.5 Sesamin Alleviates HSDFD‐Induced Hepatic Lipid Accumulation and Metabolic Abnormalities Despite Initial ADMET Concerns

Initial in silico ADMET analysis suggested that sesamin has high plasma protein binding, favourable blood–brain barrier permeability, and moderate tissue distribution (Figure S3A). Although sesamin showed relatively low predicted intestinal absorption and limited permeability in Caco‐2 and MDCK models (Figure S3B), metabolism profiling indicated a high probability of interaction with multiple cytochrome P450 enzymes, particularly CYP1A2, CYP2C19, CYP2D6, and CYP3A4 (Figure S3C). Toxicity prediction also suggested possible induction of cellular stress responses and a potential risk of drug‐induced liver injury (DILI) (Figure S3D,E).

However, subsequent in vivo experiments did not support overt hepatotoxic effects of sesamin. Sesamin supplementation did not impair liver function in mice (Figure S4). Instead, under HSDFD challenge, sesamin exerted protective effects on hepatic lipid accumulation and metabolic abnormalities.

**FIGURE 4:**
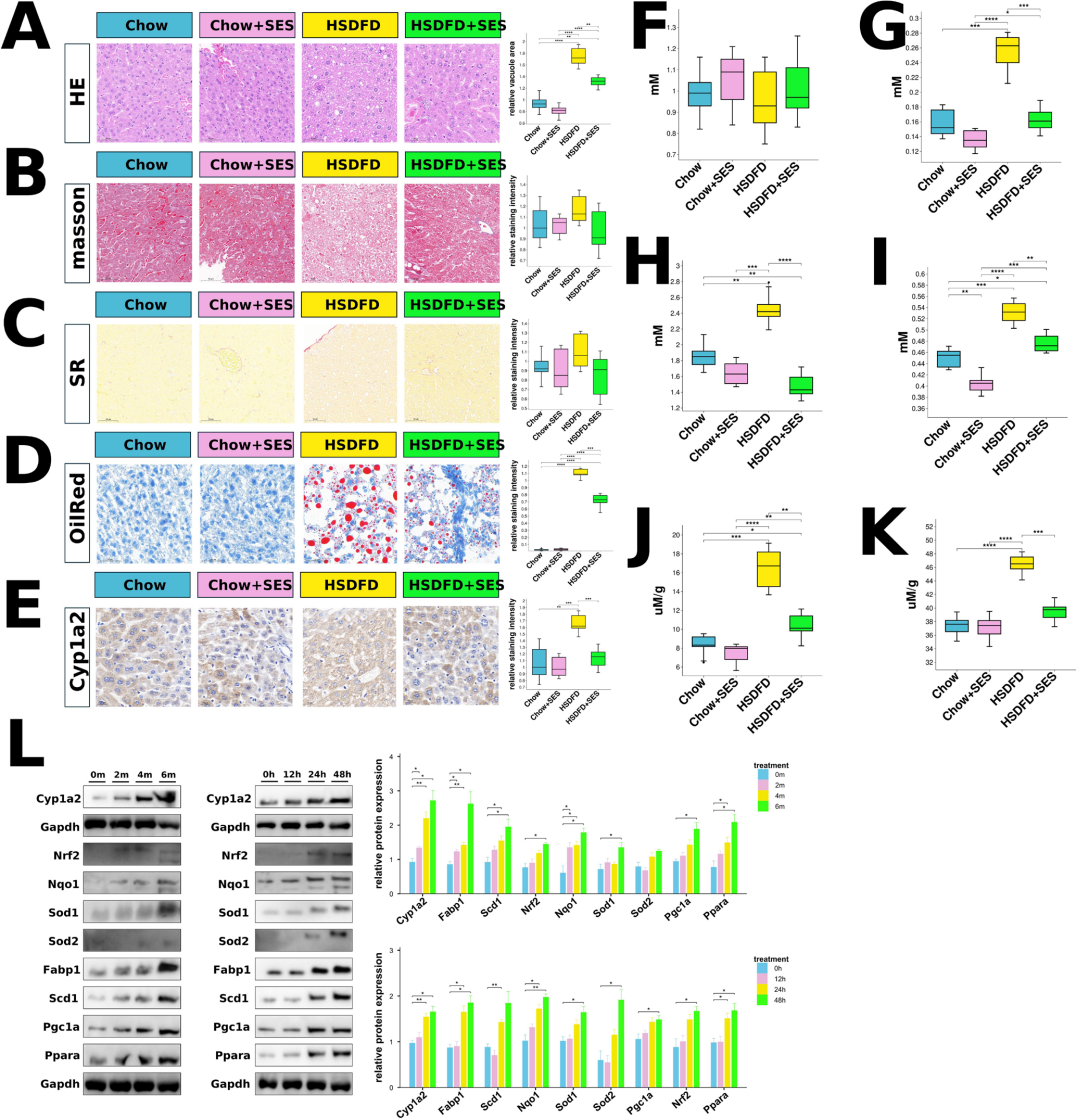
Effects of sesamin on HSDFD‐induced hepatic steatosis, lipid metabolic abnormalities, and CYP1A2‐associated oxidative stress/lipid metabolism signalling In vivo and In vitro. (A) Representative haematoxylin and eosin (H&E) staining of liver sections from mice in the Chow, Chow+SES, HSDFD, and HSDFD+SES groups, showing overall hepatic architecture and hepatocellular steatotic changes. (B) Representative Masson's trichrome staining of liver sections from each group. Blue staining indicates collagen deposition. (C) Representative Sirius Red (SR) staining of liver sections from each group, indicating hepatic fibrotic areas. (D) Representative Oil Red O staining of liver sections from each group, showing neutral lipid accumulation in the liver. (E) Representative immunohistochemical staining of Cyp1a2 in liver sections from each group. Brown staining indicates Cyp1a2‐positive signals. Relative staining intensities for panels A–E are shown on the right. (F–I) Serum lipid profiles, including HDL‐cholesterol (F), LDL‐cholesterol (G), total cholesterol (TC; H), and triglycerides (TG; I), in mice from each group. (J, K) Hepatic total cholesterol (TC; J) and triglyceride (TG; K) contents in mice from each group. (L) Western blot analysis of Cyp1a2 and oxidative stress‐ and lipid metabolism‐related proteins in liver tissues collected during HSDFD feeding and in oleic acid (OA)‐treated primary mouse hepatocytes. Left, representative immunoblots and quantification of liver tissues collected at 0, 2, 4, and 6 months during HSDFD modelling, showing the expression of Cyp1a2, Nrf2, Nqo1, Sod1, Sod2, Fabp1, Scd1, Pgc1a, and Ppara, with Gapdh as the loading control. Right, representative immunoblots and quantification of primary mouse hepatocytes exposed to OA for 0, 12, 24, and 48 h, showing the expression changes of Cyp1a2, Nrf2, Nqo1, Sod1, Sod2, Fabp1, Scd1, Pgc1a, and Ppara, with Gapdh as the loading control. These results indicate dynamic alterations of CYP1A2, oxidative stress response, and lipid metabolism‐related proteins during hepatic steatosis progression both In vivo and In vitro. Data are presented as box plots or mean ± SEM, as indicated. *n* = 8–10 mice per group for panels F–K. For panel L, data are shown as relative protein expression normalized to Gapdh. Scale bar = 100 μm. Statistical significance is indicated as *p* < 0.05, *p* < 0.01, *p* < 0.001, and *p* < 0.0001.

Histological analysis showed that HSDFD increased the number of hepatic lipid vacuoles, whereas sesamin supplementation reduced HSDFD‐induced vacuolar changes, as observed by H&E staining (Figure 4A). Consistently, Oil Red O staining revealed markedly increased lipid deposition in the livers of HSDFD‐fed mice, which was reduced by sesamin treatment (Figure 4D). In contrast, Masson's trichrome and Sirius Red staining did not reveal obvious fibrotic changes among the groups (Figure 4B,C). These findings indicate that sesamin primarily reduced hepatic lipid accumulation induced by HSDFD.

Immunohistochemical analysis further showed altered hepatic Cyp1a2 expression in response to HSDFD and sesamin treatment (Figure 4E), consistent with the predicted interaction between sesamin and CYP1A2. In addition, serum lipid profiling demonstrated that sesamin improved HSDFD‐induced lipid metabolic abnormalities. Specifically, sesamin attenuated the HSDFD‐associated increases in LDL‐C, total cholesterol (TC), and triglycerides (TG), whereas HDL‐C showed no marked change among groups (Figure 4F–I). Similar effects were observed in liver tissues, where sesamin reduced the elevated hepatic TC and TG contents induced by HSDFD (Figure 4J,K). Together, these results indicate that sesamin alleviates hepatic lipid deposition and improves systemic and hepatic lipid metabolic abnormalities in HSDFD‐fed mice.

To further examine the molecular changes during model progression, we analysed the expression of Cyp1a2, oxidative stress response‐related proteins, and lipid metabolism‐related proteins in liver tissues collected at 0, 2, 4, and 6 months during HSDFD feeding, as well as in OA‐treated primary mouse hepatocytes at 0, 12, 24, and 48 h (Figure 4L). In both the In vivo and In vitro models, Cyp1a2 expression increased progressively over time. Meanwhile, antioxidant stress response‐related proteins, including Nrf2, Nqo1, Sod1, and Sod2, were also progressively upregulated, indicating persistent and aggravated oxidative stress during model development. In parallel, lipid metabolism‐related proteins, including Fabp1, Scd1, Pgc1a, and Ppara, were concomitantly increased, suggesting progressively aggravated lipid metabolic abnormalities. Notably, at the 6‐month endpoint, hepatic GPX activity, SOD activity, and T‐AOC did not differ significantly among groups, whereas MDA levels were markedly increased in the HSDFD group and were reduced by SES treatment (Figure S5). This apparent inconsistency with the western blot results may reflect the fact that increased antioxidant protein expression represents a compensatory response to oxidative stress but does not necessarily indicate enhanced antioxidant enzyme activity at the tissue level. Overall, these findings suggest that chronic metabolic stress is accompanied by persistent oxidative burden and lipid peroxidation despite activation of antioxidant stress‐related pathways.

Importantly, the parallel increases in Cyp1a2, antioxidant stress response proteins, and lipid metabolism‐related proteins in both models suggest a close association between persistent oxidative stress and abnormal lipid metabolism.

### 3.6 Sesamin Attenuates OA‐Induced ROS Burden and Lipid Metabolic Abnormalities in Hepatocytes and Is Associated With CYP1A2‐Dependent Regulation of Antioxidant Stress Responses

**FIGURE 5:**
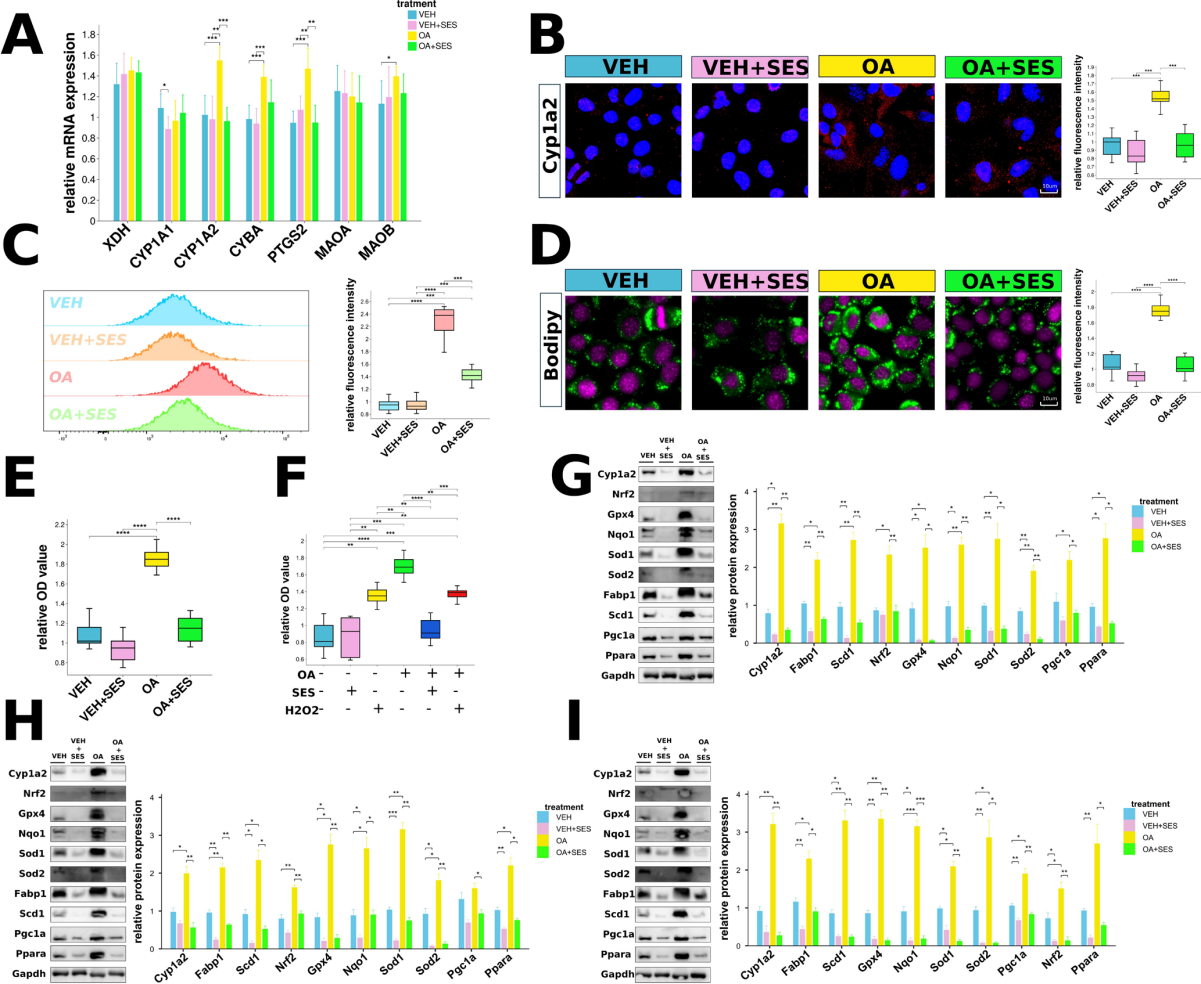
Effects of oleic acid (OA) and sesamin (SES) on oxidative stress response and lipid metabolism in hepatocytes. (A) Relative mRNA expression of oxidative stress‐related genes in AML12 cells treated with vehicle (VEH), SES, OA, or OA + SES, as determined by qPCR. (B) Representative immunofluorescence staining of Cyp1a2 (red) in AML12 cells under different treatment conditions. Nuclei were counterstained with DAPI (blue). Quantification of fluorescence intensity is shown on the right. (C) Intracellular reactive oxygen species (ROS) levels in AML12 cells under different treatment conditions, as assessed by DCFH‐DA staining and flow cytometry. Representative fluorescence histograms and quantitative analysis are shown. (D) Representative BODIPY staining of lipid droplets (green) in AML12 cells under different treatment conditions. Nuclei were counterstained with DAPI (magenta). Quantification of fluorescence intensity is shown on the right. (E) Quantitative analysis of Oil Red O staining in AML12 cells under different treatment conditions, presented as relative optical density (OD) values. (F) Quantification of Oil Red O staining in AML12 cells treated with OA, SES, and H_2_O_2_ alone or in combination, showing the effects of SES on OA‐induced lipid accumulation in the presence or absence of oxidative stress. (G) Representative western blot images and quantitative analysis of Cyp1a2, oxidative stress response‐related proteins (Nrf2, Gpx4, Nqo1, Sod1, and Sod2), and lipid metabolism‐related proteins (Fabp1, Scd1, Pgc1α, and Pparα) in AML12 mouse hepatocytes treated with VEH, VEH + SES, OA, or OA + SES. Gapdh was used as the loading control. (H) Representative western blot images and quantitative analysis of Cyp1a2, oxidative stress response‐related proteins (Nrf2, Gpx4, Nqo1, Sod1, and Sod2), and lipid metabolism‐related proteins (Fabp1, Scd1, Pgc1α, and Pparα) in primary mouse hepatocytes treated with VEH, VEH + SES, OA, or OA + SES. Gapdh was used as the loading control. (I) Representative western blot images and quantitative analysis of Cyp1a2, oxidative stress response‐related proteins (Nrf2, Gpx4, Nqo1, Sod1, and Sod2), and lipid metabolism‐related proteins (Fabp1, Scd1, Pgc1α, and Pparα) in THLE‐2 human hepatocytes treated with VEH, VEH + SES, OA, or OA + SES. Gapdh was used as the loading control.

To investigate the effects of sesamin (SES) on oleic acid (OA)‐induced hepatocellular injury, AML12 cells were treated with OA in the presence or absence of SES. qPCR analysis showed that OA altered the expression of oxidative stress‐related genes, whereas SES partially reversed these OA‐induced transcriptional changes (Figure 5A). Consistently, immunofluorescence analysis showed that Cyp1a2 protein signals were increased in OA‐treated AML12 cells and were attenuated after SES treatment (Figure 5B).

Flow cytometric analysis using DCFH‐DA further demonstrated that OA markedly increased intracellular ROS levels in AML12 cells, whereas SES reduced OA‐induced ROS accumulation (Figure 5C). In parallel, BODIPY staining and Oil Red O quantification showed that OA induced marked intracellular lipid accumulation, which was significantly reduced by SES treatment (Figure 5D,E). Moreover, the addition of H_2_O_2_ increased lipid accumulation in OA‐ and SES‐treated AML12 cells, indicating that oxidative stress promotes intracellular lipid deposition and further supporting a close association between ROS burden and lipid metabolic abnormalities (Figure 5F).

At the protein level, western blot analysis in AML12 cells, primary mouse hepatocytes, and THLE‐2 human hepatocytes consistently showed that OA increased the expression of Cyp1a2, together with the antioxidant stress response system, including Nrf2, Gpx4, Nqo1, Sod1, and Sod2 (Figure 5G–I). The activation of these antioxidant stress‐related proteins indicates the presence of persistent ROS stress under OA treatment. As CYP1A2 is a known enzyme involved in ROS generation, the OA‐induced upregulation of Cyp1a2 further suggests that increased CYP1A2 expression may contribute to intracellular ROS accumulation, thereby promoting compensatory activation of the antioxidant stress response system. At the same time, OA also increased the expression of Fabp1, Scd1, Pgc1α, and Pparα, indicating concomitant lipid metabolic dysregulation. Notably, SES treatment attenuated the OA‐induced increases in Cyp1a2, antioxidant stress response‐related proteins, and lipid metabolism‐related proteins in all three hepatocyte models (Figure 5G–I). These coordinated changes suggest a close association between oxidative stress and abnormal lipid metabolism, and further imply that SES may act on CYP1A2 to reduce ROS burden, thereby weakening compensatory activation of the antioxidant stress response system and improving lipid metabolic abnormalities.

To further compare the effects of SES with pharmacological modulation of CYP1A2, OA‐treated AML12 cells were exposed to 2,6‐Dimethylquinoline (DMQL), a known CYP1A2 inhibitor used as a positive control, or to LKY‐047 (CYP2J2 inhibitor but inactive against CYP1A2), which served as a negative control. Western blot analysis showed that treatment with DMQL was accompanied by reduced expression of Cyp1a2, antioxidant stress response‐related proteins, and lipid metabolism‐related proteins, whereas LKY‐047 showed a comparatively limited effect on these protein changes. Notably, SES produced a protein expression pattern broadly similar to that observed with CYP1A2 inhibition by DMQL (Figure S6), further supporting the possibility that SES modulates oxidative stress responses and lipid metabolic abnormalities, at least in part, through CYP1A2.

To further assess the role of CYP1A2 in this process, we next examined the effects of Cyp1a2 overexpression and Cyp1a2 knockout in AML12 cells. Western blot analysis showed that manipulation of Cyp1a2 expression significantly altered the expression patterns of Nrf2, Gpx4, Nqo1, Sod1, Sod2, Fabp1, Scd1, Pgc1α, and Pparα under the indicated treatment conditions (Figure S7A,B), further supporting the involvement of CYP1A2 in coordinating antioxidant stress responses and lipid metabolism‐related protein expression in hepatocytes.

Taken together, these findings indicate that OA induces persistent ROS stress, accompanied by upregulation of CYP1A2, activation of the antioxidant stress response system, and increased expression of lipid metabolism‐related proteins. Given that CYP1A2 is a known contributor to ROS generation, SES may alleviate ROS burden, at least in part, through modulation of CYP1A2, thereby weakening antioxidant stress system activation and improving lipid metabolic abnormalities.

### 3.7 Sesamin Exhibits Minimal Allosteric Effects on CYP1A2 Structure and Dynamics

**FIGURE 6:**
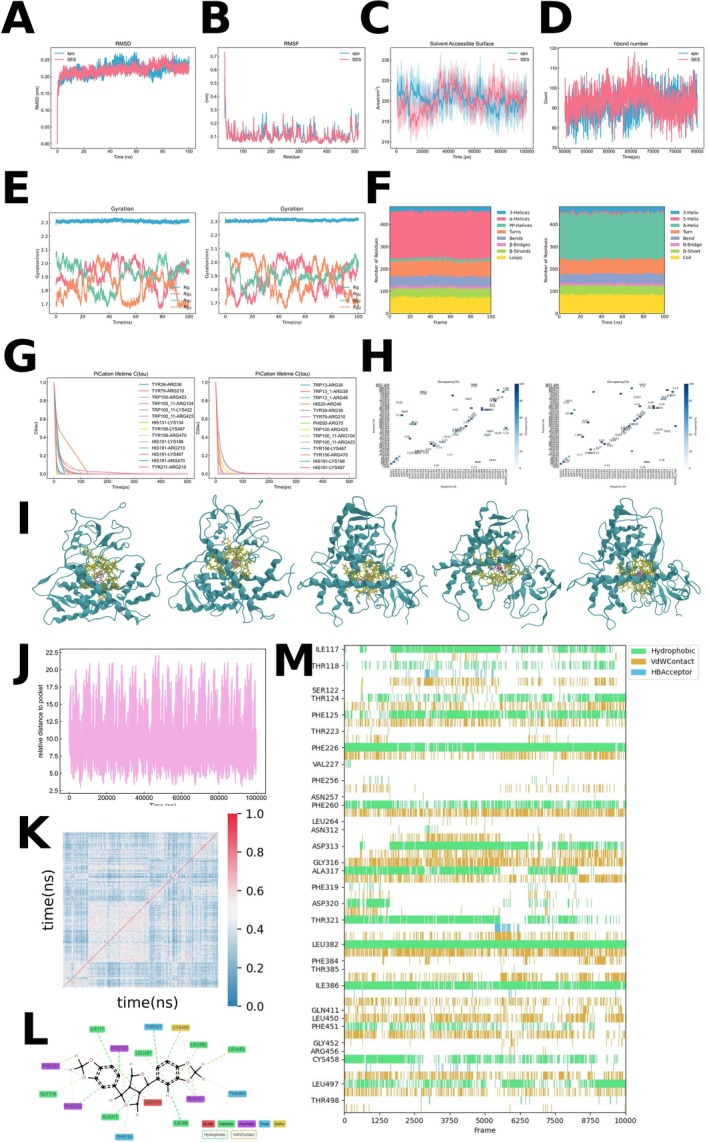
Molecular Dynamics Simulation Analysis of Sesamin (SES) Interaction with CYP1A2 (A) Root Mean Square Deviation (RMSD) of CYP1A2 backbone atoms with (SES) and without (apo) sesamin over 100 ns simulation. (B) Root Mean Square Fluctuation (RMSF) of CYP1A2 residues with and without sesamin. (C) Solvent Accessible Surface Area (SASA) of CYP1A2 with and without sesamin during the simulation. (D) Number of hydrogen bonds between sesamin and CYP1A2 over time. (E) Radius of gyration (Rg) for CYP1A2 with and without sesamin, showing overall protein compactness. (F) DSSP (Define Secondary Structure of Proteins) analysis showing the evolution of CYP1A2 secondary structure elements over time with and without sesamin. (G) Pi‐cation interaction lifetimes between sesamin and various CYP1A2 residues. (H) Salt bridge occupancy between specific residue pairs in CYP1A2 with and without sesamin. (I) Snapshots of sesamin binding to CYP1A2 at 0, 25, 50, 75, and 100 ns of the simulation. (J) Time evolution of the relative distance between sesamin and key pocket residues of CYP1A2 during the MD simulation. (K) Tanimoto similarity matrix for sesamin, assessing the similarity of sesamin molecular fingerprints (bitvectors) over the course of the dynamics, reflecting conformational changes of sesamin during the simulation. (L) 2D interaction diagram showing the weighted interactions between sesamin and CYP1A2 residues. (M) Time‐dependent analysis of different types of interactions between sesamin and specific CYP1A2 residues throughout the MD simulation, categorized as hydrophobic, van der Waals contacts, and hydrogen bond acceptors.

Molecular dynamics simulations revealed that sesamin binding to CYP1A2 did not induce significant allosteric effects compared to the apo state. This was evidenced by highly similar RMSD, RMSF, solvent accessible surface area (SASA), hydrogen bond numbers, and gyration radius between apo and sesamin‐bound states (Figure 6A–E). The DSSP analysis and π‐cation lifetime C (tau) showed minimal differences, and the salt bridge occupancy remained consistent between the apo and sesamin‐bound states, collectively indicating preservation of secondary structure elements and key interactions (Figure 6F–H).

Sesamin maintained a stable binding pose within the CYP1A2 pocket throughout the simulation, exhibiting periodic motions (Figure 6I,J). The Animoto similarity matrix for sesamin reflected these conformational changes over time (Figure 6K). Analysis of sesamin‐CYP1A2 interactions revealed predominant hydrophobic and van der Waals contacts, with residues ILE386, THR321, ILE117, and PHE125 showing strong interactions (Figure 6L). Time‐frame analysis demonstrated persistent interactions with PHE226, PHE260, ALA317, LEU382, and ILE386 throughout the simulation, while THR118, THR321, and CYS458 intermittently acted as hydrogen bond acceptors (Figure 6M).

Conformational evolution studies using various dimensionality reduction techniques provided insights into the subtle effects of sesamin binding. Dihedral angle‐based PCA revealed that the apo state formed two discrete conformational clusters, indicating multiple dihedral angle switches. In contrast, the sesamin‐bound state showed a more continuous, compact cluster, suggesting suppression of dihedral angle changes (Figure S8A). Coordinate‐based t‐SNE, UMAP, and tICA analyses displayed continuous manifolds for both states, evolving over time (Figure S8B–D).

Free energy landscape (FEL) analysis showed three evenly distributed energy wells for the apo state, while the sesamin‐bound state formed one major and two minor energy wells with increased depth. The lowest energy conformation was reached at 32.95 ns in the sesamin‐bound state (Figure S8E,F).

Residue distance‐based analyses, including the residue distance correlation matrix (RDCM), time occupancy of contact matrix, and dynamic cross‐correlation matrix (DCCM), demonstrated largely consistent patterns between apo and sesamin‐bound states (Figure S9A–C). However, the Pearson correlation coefficient matrix of residue distances over time showed increased positive correlations in certain regions for the sesamin‐bound state, accompanied by reorganization of residue branches in hierarchical clustering (Figure S9D,E). PCA based on RDCM revealed concentrated conformational distributions for both states, further confirming the subtle nature of conformational changes induced by sesamin binding (Figure S9F).

Network analysis of the shortest and second‐shortest path networks showed that sesamin binding increased network size and degree, with some residue communities disappearing and others being reconstructed. Specifically, sesamin enhanced connections between helices and loops in the outer regions of CYP1A2's core (Figure S10A,B).

DCCM analysis based on MD trajectories revealed fragmented residue correlations in both apo and sesamin‐bound states. While sesamin binding did not significantly alter the overall correlation pattern, it further increased fragmentation, indicating localized, subtle conformational changes (Figure S10C,D).

In conclusion, these comprehensive analyses demonstrate that sesamin binding to CYP1A2 induces minimal allosteric effects, primarily causing localized, subtle conformational changes without significantly altering the protein's global structure or dynamics.

### 3.8 Molecular Dynamics and Experimental Validation Support a Direct Interaction Between Sesamin and CYP1A2

**FIGURE 7:**
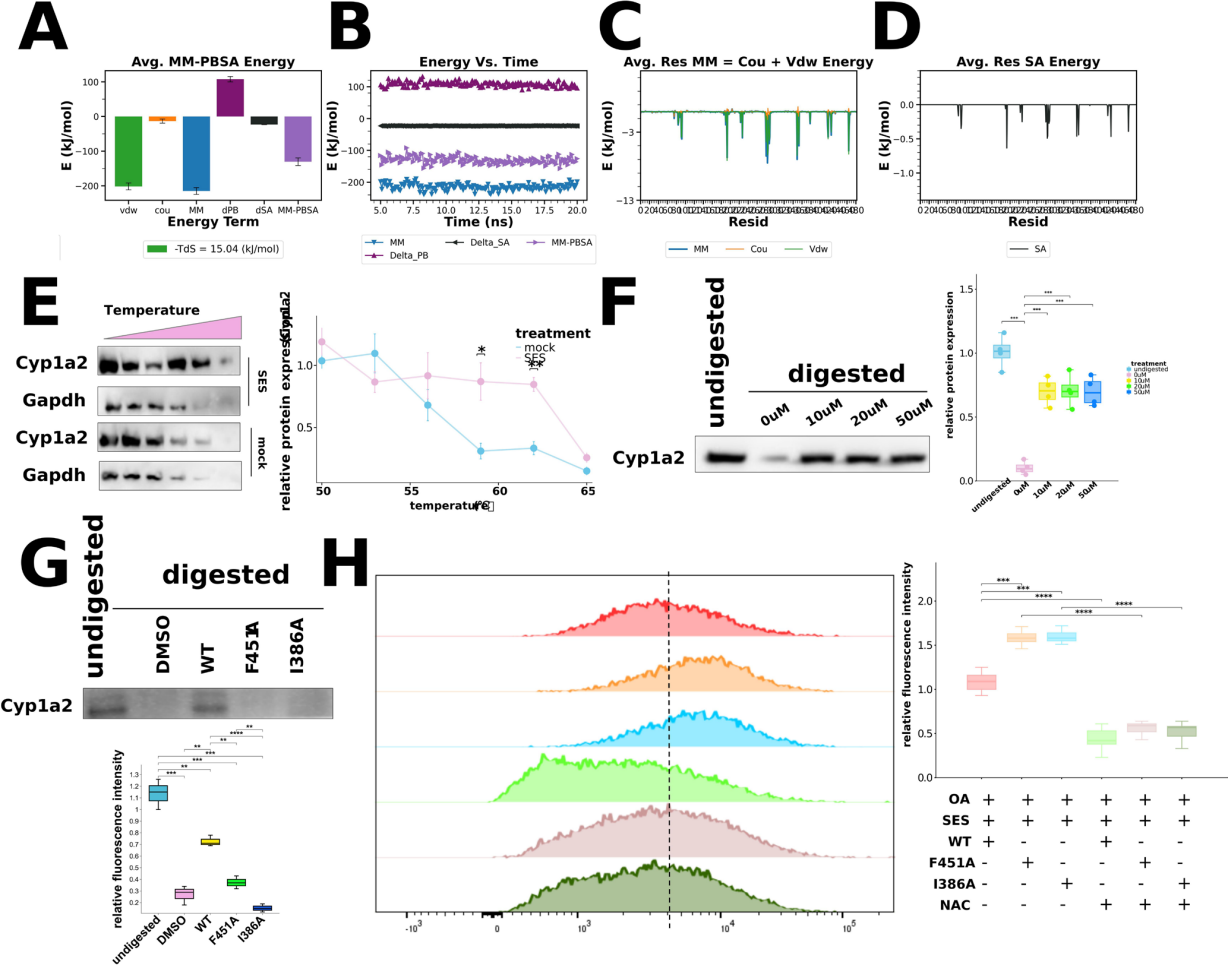
Energetic and Experimental Analysis of Sesamin (SES) Interaction with CYP1A2 (A) Avg. MM‐PBSA Energy: Contributions of different components to the MM‐PBSA energy, including van der Waals (vdw), Coulombic (Cou), MM, Poisson‐Boltzmann (PB), Surface Area (SA), and total MM‐PBSA energy. (B) Energy vs. Time: Time evolution of MM, SA, PB, and total MM‐PBSA energy components during the simulation. (C) Avg. Res MM + Cou + Vdw Energy: Residue‐wise decomposition of the average MM energy contribution, including Coulombic and van der Waals components. (D) Avg. Res SA Energy: Residue‐wise decomposition of the average SA energy contribution. (E) Cellular thermal shift assay (CETSA) showing protein stability with and without SES treatment at different temperatures. (F) Drug affinity responsive target stability (DARTS) assay showing the effects of different concentrations of sesamin (SES) on the protease stability of CYP1A2 protein. Representative immunoblot images and corresponding quantification are shown. (G) DARTS assay comparing the protease stability of WT CYP1A2 and the F451A/I386A CYP1A2 mutant. Representative immunoblot images and quantitative analysis are shown. (H) BODIPY flow cytometric analysis of lipid accumulation in AML12 cells expressing WT CYP1A2 or F451A/I386A CYP1A2 under the indicated treatments. The F451A/I386A mutation partially attenuated the protective effect of SES against OA‐induced lipid accumulation in AML12 cells, whereas treatment with the antioxidant N‐acetyl‐L‐cysteine (NAC) reduced lipid deposition in both WT‐ and F451A/I386A CYP1A2‐expressing AML12 cells.

To further characterize the interaction between sesamin (SES) and CYP1A2, we performed molecular dynamics simulation followed by MM‐PBSA analysis of the SES–CYP1A2 complex. The calculated binding free energy was −130.498 ± 10.8952 kJ/mol, indicating a favourable and stable interaction (Figure 7A,B; Figure S11). Energy component analysis showed that van der Waals interactions and the overall molecular mechanics (MM) term made major contributions to the binding free energy, supporting the presence of a stable SES–CYP1A2 binding mode. The time evolution of the major energy components further indicated that the complex remained stable throughout the simulation.

Residue‐level free energy decomposition further identified multiple amino acid residues that contributed to SES binding (Figure 7C,D; Figure S11). Based on the docking and simulation results, I386 and F451 were selected for subsequent experimental validation.

To verify direct target engagement experimentally, we first performed a cellular thermal shift assay (CETSA). SES treatment increased the thermal stability of CYP1A2 over a range of temperatures, indicating direct binding of SES to CYP1A2 in cells (Figure 7E). We then conducted a drug affinity responsive target stability (DARTS) assay and found that SES increased the protease resistance of CYP1A2 in a concentration‐dependent manner (Figure 7F), further supporting direct interaction between SES and CYP1A2.

To evaluate the importance of the predicted binding residues, we next compared the protease stability of WT CYP1A2 and the F451A/I386A mutant in the DARTS assay. SES increased the stability of WT CYP1A2 against proteolysis, whereas this stabilizing effect was markedly weakened in the F451A/I386A mutant (Figure 7G). These results support the notion that F451 and I386 are important residues for SES binding to CYP1A2.

We then examined the functional consequence of disrupting this interaction using BODIPY‐based flow cytometry in AML12 cells expressing WT CYP1A2 or the F451A/I386A mutant. SES reduced OA‐induced lipid accumulation in cells expressing WT CYP1A2, whereas the F451A/I386A mutation partially attenuated this protective effect (Figure 7H). In contrast, treatment with the antioxidant N‐acetyl‐L‐cysteine (NAC) reduced lipid deposition in both WT‐ and F451A/I386A‐expressing cells. These findings suggest that the protective effect of SES on OA‐induced lipid accumulation depends, at least in part, on its interaction with CYP1A2, while direct scavenging of ROS by NAC can bypass the impaired SES–CYP1A2 interaction.

Taken together, the molecular dynamics, MM‐PBSA, CETSA, and DARTS results consistently support a direct and stable interaction between SES and CYP1A2. Functional analysis further indicates that I386 and F451 are important for this interaction and that disruption of these residues partially weakens the ability of SES to reduce OA‐induced lipid accumulation. These results support a model in which SES interacts with CYP1A2 to modulate ROS‐related lipid metabolic abnormalities in hepatocytes.

## 4 Discussion

Our study provides novel insights into the molecular mechanisms underlying sesamin's hepatoprotective effects in the context of high‐sugar, high‐fat diet (HSDFD)‐induced metabolic disturbances. We demonstrate that HSDFD triggers a significant redistribution of hepatocyte subpopulations, characterized by widespread high abundance of CYP1A2, a member of the cytochrome P450 family. This shift is accompanied by alterations in fatty acid metabolism and xenobiotic metabolism pathways, suggesting a complex interplay between dietary factors and hepatic metabolic processes [23, 24].

The observed changes in hepatocyte subpopulations and their metabolic profiles underscore the liver's adaptive response to HSDFD‐induced stress. The high abundance of CYP1A2 across these hepatocyte subpopulations is particularly noteworthy, as it implies a central role for this enzyme in mediating the liver's response to metabolic challenges [25, 26, 27, 28, 29]. This finding aligns with previous studies highlighting the importance of cytochrome P450 enzymes in lipid homeostasis and xenobiotic metabolism, but offers a more nuanced understanding of their involvement in diet‐induced metabolic disturbances [26, 30, 31, 32, 33]. The differential expression of CYP1A2 among various hepatocyte subpopulations suggests a complex and heterogeneous response of liver cells to HSDFD, potentially indicating specialized roles for different hepatocyte subsets in managing metabolic stress.

Sesamin has been previously reported to possess various beneficial effects, including antioxidant, anti‐inflammatory, and lipid‐lowering properties [11, 12, 13]. Our bulk RNA sequencing analysis, consistent with earlier studies, indicates that sesamin supplementation potentially modulates fatty acid metabolism pathways and oxidative stress responses, particularly fatty acid β‐oxidation. These observations suggest a multifaceted action of sesamin in ameliorating HSDFD‐induced hepatic dysfunction, prompting us to investigate its specific molecular targets.

Through an innovative approach utilizing the AlphaFold database, we identified CYP1A2 as a potential direct target of sesamin in mammals [22]. Our findings demonstrate that sesamin's beneficial effects on hepatic lipid metabolism are mediated through the regulation of reactive oxygen species (ROS), corroborating previous research on sesamin's antioxidant properties [13, 34]. This mechanism of action provides a plausible explanation for sesamin's broad spectrum of beneficial effects, as ROS play a crucial role in various cellular processes and signalling pathways.

Intriguingly, our analysis revealed species‐specific interactions between sesamin and CYP1A2. While the binding mode in rats and mice showed similarities, it differed from that observed in humans. This species heterogeneity in sesamin‐CYP1A2 interactions highlights the importance of considering interspecies differences in drug‐target interactions and underscores the need for caution when extrapolating results from animal models to humans [35].

Molecular dynamics simulations and MM‐PBSA analysis further confirmed the stable binding of sesamin to CYP1A2, albeit with minimal overall allosteric effects on the protein structure. This suggests that sesamin's modulatory effects on CYP1A2 activity may be achieved through subtle conformational changes or by influencing the enzyme's interactions with other cellular components, rather than through large‐scale structural alterations.

Based on molecular docking, dynamics simulations, and MM‐PBSA residue decomposition, we identified F451 and I386 as key residues involved in sesamin binding and its regulation of CYP1A2‐mediated ROS production. These computational predictions were experimentally validated through DARTS assays and BODIPY flow cytometry rescue experiments, providing strong evidence for the functional importance of these residues in sesamin's hepatoprotective effects.

Our findings open up several promising avenues for future research. A key area of investigation should be the precise mechanisms through which sesamin‐CYP1A2 interactions modulate ROS production and lipid metabolism [36, 37]. Understanding these molecular pathways in greater detail could provide valuable insights into the regulation of hepatic metabolism and potentially reveal new therapeutic targets. Additionally, given the complex nature of metabolic disorders, it would be worthwhile to explore potential synergistic effects between sesamin and other natural compounds or pharmaceuticals that target hepatic metabolism. Such combinations might offer more potent or comprehensive approaches to addressing liver dysfunction [19, 38, 39]. Furthermore, while our study focused on HSDFD‐induced hepatic disturbances, the therapeutic potential of sesamin or sesamin‐derived compounds may extend to various other liver disorders [40, 41, 42]. Investigating their efficacy in conditions such as non‐alcoholic fatty liver disease, cirrhosis, or drug‐induced liver injury could significantly broaden the clinical applications of these compounds.

This study has several limitations that should be acknowledged. First, although CYP1A2 was consistently upregulated in our dietary and cellular models, the biological meaning of this induction remains to be fully clarified. Given the dual role of CYP1A2 in xenobiotic metabolism and ROS generation, its upregulation may represent an adaptive response at early stages of metabolic stress, aimed at maintaining hepatic homeostasis, but under sustained lipid overload it may also contribute to excessive ROS accumulation and thereby become maladaptive. Our current data support an association between increased CYP1A2 expression, activation of the antioxidant stress response system, and lipid metabolic abnormalities, but they do not definitively distinguish whether CYP1A2 induction is initially protective and later detrimental during chronic metabolic stress. Second, although sesamin showed consistent protective effects in mouse models and also attenuated OA‐induced oxidative stress responses and lipid metabolic abnormalities in the human hepatocyte line THLE‐2, these findings should not be overinterpreted as direct evidence of clinical efficacy in humans. Species‐specific differences in CYP1A2 expression, catalytic activity, hepatic metabolism, and systemic pharmacokinetics may substantially influence sesamin responsiveness in vivo. In addition, THLE‐2 cells provide only preliminary human‐relevant evidence and cannot fully recapitulate the complexity of human liver physiology. Therefore, the translational relevance of our findings remains to be further validated in primary human hepatocytes, human liver organoid systems, and ultimately in well‐designed clinical studies.

Our study integrates advanced computational methods with experimental validation, revealing CYP1A2 as a key target of sesamin in ameliorating HSHFD‐induced hepatic dysfunction. By elucidating specific residues involved and demonstrating functional relevance in ROS‐mediated lipid metabolism, we provide a foundation for future metabolic liver disorder therapies. This research highlights natural compounds' potential in addressing complex metabolic disturbances, despite limitations in animal models.

## Author Contributions

**Xiliang Zhu:** conceptualization (equal), investigation (equal), supervision (equal), validation (equal), writing – original draft (equal). **Jie Yan:** conceptualization (equal), data curation (equal), resources (equal), software (equal), validation (equal). **Qi Liu:** conceptualization (equal), data curation (equal), formal analysis (equal), supervision (equal), validation (equal), writing – original draft (equal). **Zhaoyun Cheng:** investigation (equal), project administration (equal), software (equal), supervision (equal), writing – original draft (equal). **Yi Luo:** data curation (equal), formal analysis (equal), validation (equal), visualization (equal), writing – original draft (equal).

## Funding

This work was supported by the National Natural Science Foundation of China Grant (No. 82200434).

## Consent

The authors have nothing to report.

## Conflicts of Interest

The authors declare no conflicts of interest.

## Supporting information


**Figure S1:** Single‐cell analysis of hepatocyte subpopulations under chow and high‐sugar, high‐fat diet (HSHFD) conditions. (A) Metabolic activity scores across different hepatocyte subpopulations. (B) Heatmap showing activity of various metabolic pathways in different hepatocyte subpopulations. (C) UMAP plots illustrating fatty acid elongation activity in hepatocyte subpopulations under chow and HSHFD conditions. (D) UMAP plots showing fatty acid biosynthesis activity in hepatocyte subpopulations under chow and HSHFD conditions. (E) UMAP plots depicting fatty acid degradation activity in hepatocyte subpopulations under chow and HSHFD conditions. (F) UMAP plots showing activity of xenobiotic metabolism by cytochrome P450 in hepatocyte subpopulations under chow and HSHFD conditions. (G) Mapping of hepatocyte subpopulations from chow and HSHFD conditions to human liver single‐cell atlas. (H) Proportion of hepatocyte subpopulations mapped to different human hepatocyte subtypes under chow and HSHFD conditions. (I) UMAP plots showing how different mouse hepatocyte subpopulations map to human liver atlas hepatocyte subtypes.
**Figure S2:** Enrichment of Lipid Metabolism Pathways in Different Hepatic Cell Subpopulations from the HPA Database (A) UMAP plot depicting the clustering of different hepatic cell subpopulations based on single‐cell RNA‐seq data from human liver, with a bar chart indicating the number of cells in each subpopulation. The distinct clusters represent various hepatic cell types, including hepatocytes, T‐cells, Kupffer cells, and others. (B) UMAP plot illustrating the enrichment of the fatty acid biosynthesis pathway across different hepatic cell subpopulations, highlighting specific clusters with elevated pathway activity. (C) UMAP plot showing the enrichment of the fatty acid elongation pathway within various hepatic cell subpopulations, indicating the differential involvement of this pathway among different cell types. (D) UMAP plot displaying the enrichment of the fatty acid degradation pathway in different hepatic cell subpopulations, with certain clusters exhibiting higher activity levels.
**Figure S3:** ADMET Analysis and Toxicity Profiling of Sesamin (SES) (A) Physicochemical properties and drug‐likeness parameters of SES. The chart displays values for various properties including Fu (fraction unbound), PPB (plasma protein binding), BBB (blood–brain barrier permeability), BCBP (breast cancer resistance protein), MRP1 (multidrug resistance‐associated protein) (1), BSEP (bile salt export pump), OATP1B1 and OATP1B3 (organic anion transporting polypeptides), and logD (distribution coefficient). (B) Absorption profile of SES across different cell models. The chart shows permeability coefficients (in log scale) for MDCK (Madin‐Darby Canine Kidney) cells, Caco‐2 cells, PAMPA (Parallel Artificial Membrane Permeability Assay), Pgp substrate and inhibition potential, HIA (Human Intestinal Absorption), and F20, F30, F50 (oral bioavailability at 20, 30, and 50 mg/kg doses). (C) Metabolism profile of SES, illustrating its interaction with various cytochrome P450 enzymes. The radar chart displays substrate and inhibition potentials for CYP1A2, CYP2C9, CYP2C19, CYP2D6, CYP3A4, and other CYP isoforms. (D) Nuclear receptor interactions of SES. The radar chart shows the potential interactions of SES with various nuclear receptors including PXR, CAR, AhR, and others, which play crucial roles in xenobiotic metabolism and toxicity. (E) Toxicity profile of SES based on various in silico and In vitro assays. The radar chart displays predicted outcomes for different toxicity endpoints including carcinogenicity, mutagenicity, hepatotoxicity, cardiotoxicity (hERG inhibition), and specific cell line toxicities (A549, HepG2, etc.).
**Figure S4:** Changes in Liver Function Parameters in Mice Under Different Dietary Conditions (A) Serum albumin (ALB) levels (g/L) across different dietary groups. (B) Serum alkaline phosphatase (ALP) activity (U/L) across different dietary groups. (C) Serum alanine aminotransferase (ALT) activity (U/L) across different dietary groups. (D) Serum aspartate aminotransferase (AST) activity (U/L) across different dietary groups. (E) Serum total protein (TP) levels (g/L) across different dietary groups.
**Figure S5:** Changes in Hepatic Oxidative Stress Parameters in Mice Under Different Dietary Conditions (A) Glutathione peroxidase (GPX) activity (U/L) in liver tissue across different dietary groups. (B) Superoxide dismutase (SOD) activity (U/L) in liver tissue across different dietary groups. (C) Total antioxidant capacity (T‐AOC) (mM/g) in liver tissue across different dietary groups. (D) Malondialdehyde (MDA) levels (mM/mg), a marker of lipid peroxidation, in liver tissue across different dietary groups.
Figure.S6: Effects of sesamin, the CYP1A2 inhibitor 2,6‐Dimethylquinoline (DMQL), and the negative control compound LKY‐047 on CYP1A2, antioxidant stress response‐related proteins, and lipid metabolism‐related proteins in OA‐treated AML12 cells.
**Figure S7:** Effects of CYP1A2 overexpression or knockout on oxidative stress response‐ and lipid metabolism‐related proteins in AML12 hepatocytes. (A) Representative western blot images and quantitative analysis of Cyp1a2, oxidative stress response‐related proteins (Nrf2, Gpx4, Nqo1, Sod1, and Sod2), and lipid metabolism‐related proteins (Fabp1, Scd1, Pgc1α, and Pparα) in AML12 cells with Cyp1a2 overexpression under the indicated treatment conditions. Gapdh was used as the loading control. (B) Representative western blot images and quantitative analysis of Cyp1a2, oxidative stress response‐related proteins (Nrf2, Gpx4, Nqo1, Sod1, and Sod2), and lipid metabolism‐related proteins (Fabp1, Scd1, Pgc1α, and Pparα) in AML12 cells with Cyp1a2 knockout under the indicated treatment conditions. Gapdh was used as the loading control.
**Figure S8:** Conformational Analysis and Energy Landscape of CYP1A2 in Apo and Sesamin‐Bound States (A) Principal Component Analysis (PCA) based on dihedral angles of CYP1A2. Left: Apo state. Right: Sesamin (SES)‐bound state. The colour gradient represents simulation time progression. (B) t‐SNE (t‐Distributed Stochastic Neighbour Embedding) analysis based on atomic coordinates of CYP1A2. Left: Apo state. Right: SES‐bound state. The colour gradient indicates simulation time. (C) UMAP (Uniform Manifold Approximation and Projection) analysis based on atomic coordinates of CYP1A2. Left: Apo state. Right: SES‐bound state. The colour gradient shows simulation time progression. (D) tICA (time‐lagged Independent Component Analysis) based on atomic coordinates of CYP1A2. Left: Apo state. Right: SES‐bound state. The colour gradient represents simulation time. (E) Free Energy Landscape (FEL) of CYP1A2 in the Apo state. Left: Gibbs energy landscape plotted against the first two principal components. Right: Scatter plot showing the time required to reach energy minima (conformational traps). Colour gradient indicates simulation time. (F) Free Energy Landscape (FEL) of CYP1A2 in the SES‐bound state. Left: Gibbs energy landscape plotted against the first two principal components. Right: Scatter plot showing the time required to reach energy minima. Colour gradient represents simulation time.
**Figure S9:** Residue Distance‐Based Correlation Analysis of CYP1A2 in Apo and Sesamin‐Bound States (A) Average distance matrices between residues. Left: Apo state. Right: Sesamin (SES)‐bound state. Colour scale indicates distance in nm. (B) Time occupancy of contact matrices. Left: Apo state. Right: SES‐bound state. Colour scale represents the fraction of simulation time that residues are in contact. (C) Dynamic Cross‐Correlation Matrices (DCCM) based on Residue Distance Correlation Matrices (RDCM). Left: Apo state. Right: SES‐bound state. Colour scale indicates the degree of correlated motion between residues. (D) Pearson correlation matrices of residue distances with simulation time. Left: Apo state. Right: SES‐bound state. Colour scale shows the strength and direction of correlation. (E) Hierarchical clustering dendrograms based on residue distances. Left: Apo state. Right: SES‐bound state. Colours represent different clusters of residues with similar distance patterns. (F) Principal Component Analysis (PCA) of Residue Distance Correlation Matrices (RDCM). Left: Apo state. Right: SES‐bound state. Colour gradient indicates simulation time progression.
**Figure S10:** Structural and Dynamic Analysis of CYP1A2 in Apo and Sesamin‐Bound States (A) Residue shortest path networks of CYP1A2 in apo (left) and sesamin‐bound (right) states. The protein structure is shown in grey ribbon representation, with coloured spheres and lines representing key residues and their connections in the shortest path network. Adjacent to each protein structure is the corresponding network diagram. (B) Residue second shortest path networks of CYP1A2 in apo (left) and sesamin‐bound (right) states. Similar to panel A, but showing alternative communication pathways within the protein. (C) Dynamic Cross‐Correlation Matrix (DCCM) analysis of CYP1A2 in the apo state. Left: DCCM plot showing correlations between residue motions. Right: Three‐dimensional representations of the protein showing correlated motions at different hierarchical levels, from local (left) to global (right) correlations. (D) DCCM analysis of CYP1A2 in the sesamin‐bound state. Arranged similarly to panel C, showing how sesamin binding affects residue motion correlations throughout the protein.
**Figure S11:** Molecular Dynamics Analysis of Sesamin (SES) Interaction with CYP1A2 (A) MM Energy vs. Time: Time evolution of van der Waals (Vdw), Coulombic (Cou), and total Molecular Mechanics (MM) energy components during the simulation. (B) PB Energy vs. Time: Time evolution of Poisson‐Boltzmann (PB) energy components, including complex (PB_Com), ligand (PB_Lig), protein (PB_Pro), and total PB energy (Delta_PB). (C) SA Energy vs. Time: Time evolution of Surface Area (SA) energy components, including complex (SA_Com), ligand (SA_Lig), protein (SA_Pro), and total SA energy (Delta_SA). (D) Avg. Res PB Energy: Residue‐wise decomposition of the average PB energy contribution.

## Data Availability

All data generated or analysed during this study are included in this published article and its Supporting Information files, or are available from the corresponding author on reasonable request.
