# Supplementary material for: Sesamin Ameliorates High‐Sugar, High‐Fat Diet‐Induced Hepatic Dysfunction via CYP1A2‐Mediated Regulation of Lipid Metabolism and Oxidative Stress
Source: J Cell Mol Med. 2026 Jul 17;30(14):e71274. doi: 10.1111/jcmm.71274 (PMC13378100; doi:10.1111/jcmm.71274)
Supplement: Supplementary file 1 — Figure S1: Single‐cell analysis of hepatocyte subpopulations under chow and high‐sugar, high‐fat diet (HSHFD) conditions. (A) Metabolic activity scores across different hepatocyte subpopulations. (B) Heatmap showing activity of various metabolic pathways in different hepatocyte subpopulations. (C) UMAP plots illustrating fatty acid elongation activity in hepatocyte subpopulations under chow and HSHFD conditions. (D) UMAP plots showing fatty acid biosynthesis activity in hepatocyte subpopulations under chow and HSHFD conditions. (E) UMAP plots depicting fatty acid degradation activity in hepatocyte subpopulations under chow and HSHFD conditions. (F) UMAP plots showing activity of xenobiotic metabolism by cytochrome P450 in hepatocyte subpopulations under chow and HSHFD conditions. (G) Mapping of hepatocyte subpopulations from chow and HSHFD conditions to human liver single‐cell atlas. (H) Proportion of hepatocyte subpopulations mapped to different human hepatocyte subtypes under chow and HSHFD conditions. (I) UMAP plots showing how different mouse hepatocyte subpopulations map to human liver atlas hepatocyte subtypes. Figure S2: Enrichment of Lipid Metabolism Pathways in Different Hepatic Cell Subpopulations from the HPA Database (A) UMAP plot depicting the clustering of different hepatic cell subpopulations based on single‐cell RNA‐seq data from human liver, with a bar chart indicating the number of cells in each subpopulation. The distinct clusters represent various hepatic cell types, including hepatocytes, T‐cells, Kupffer cells, and others. (B) UMAP plot illustrating the enrichment of the fatty acid biosynthesis pathway across different hepatic cell subpopulations, highlighting specific clusters with elevated pathway activity. (C) UMAP plot showing the enrichment of the fatty acid elongation pathway within various hepatic cell subpopulations, indicating the differential involvement of this pathway among different cell types. (D) UMAP plot displaying the enrichme [file JCMM-30-e71274-s001.zip › Supplement.docx]

**Supplementary Materials**


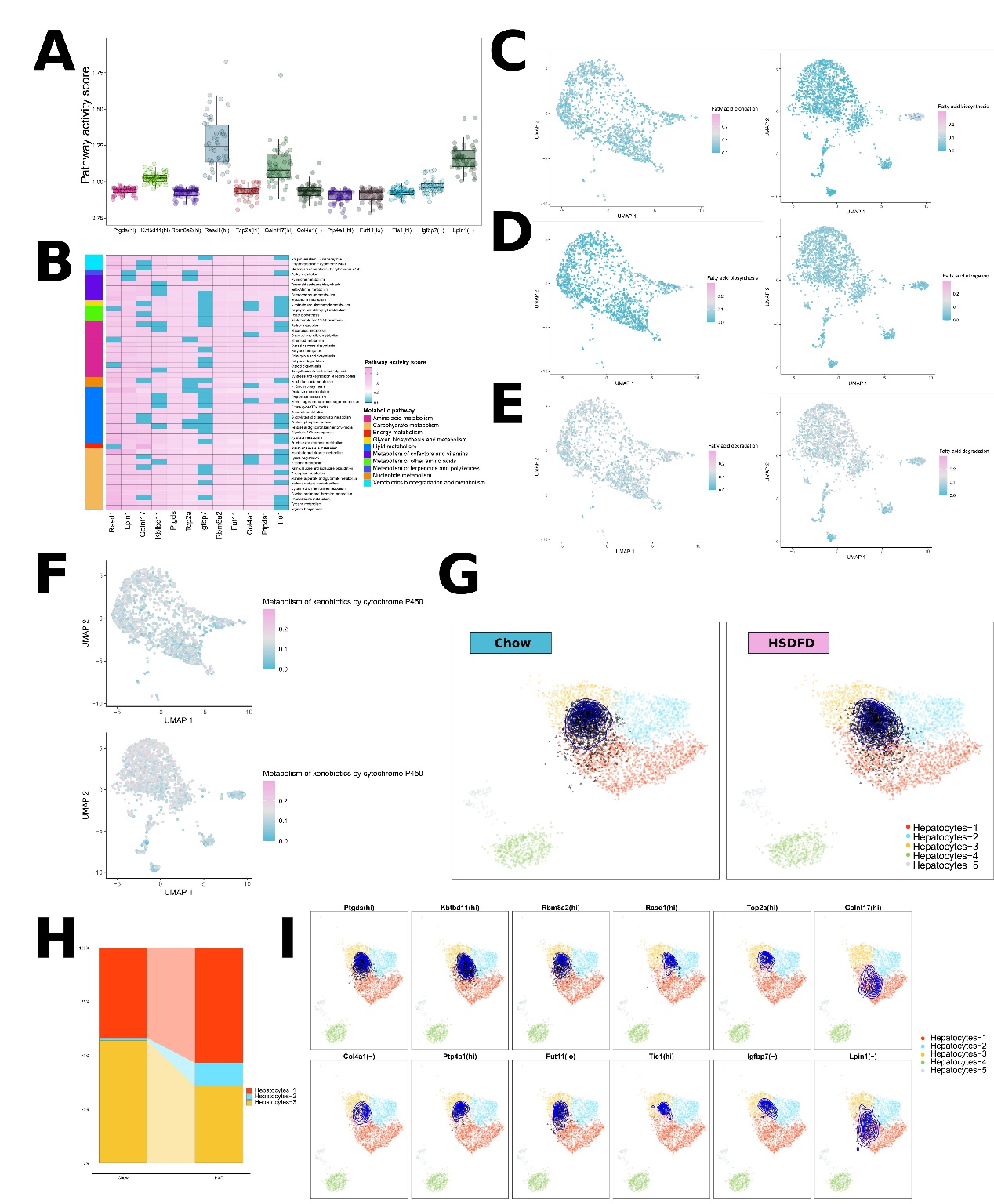


Supplementary Figure 1: Single-cell analysis of hepatocyte subpopulations under chow and high-sugar, high-fat diet (HSHFD) conditions.(A) Metabolic activity scores across different hepatocyte subpopulations.(B) Heatmap showing activity of various metabolic pathways in different hepatocyte subpopulations.(C) UMAP plots illustrating fatty acid elongation activity in hepatocyte subpopulations under chow and HSHFD conditions.(D) UMAP plots showing fatty acid biosynthesis activity in hepatocyte subpopulations under chow and HSHFD conditions.(E) UMAP plots depicting fatty acid degradation activity in hepatocyte subpopulations under chow and HSHFD conditions.(F) UMAP plots showing activity of xenobiotic metabolism by cytochrome P450 in hepatocyte subpopulations under chow and HSHFD conditions.(G) Mapping of hepatocyte subpopulations from chow and HSHFD conditions to human liver single-cell atlas.(H) Proportion of hepatocyte subpopulations mapped to different human hepatocyte subtypes under chow and HSHFD conditions.(I) UMAP plots showing how different mouse hepatocyte subpopulations map to human liver atlas hepatocyte subtypes.

**
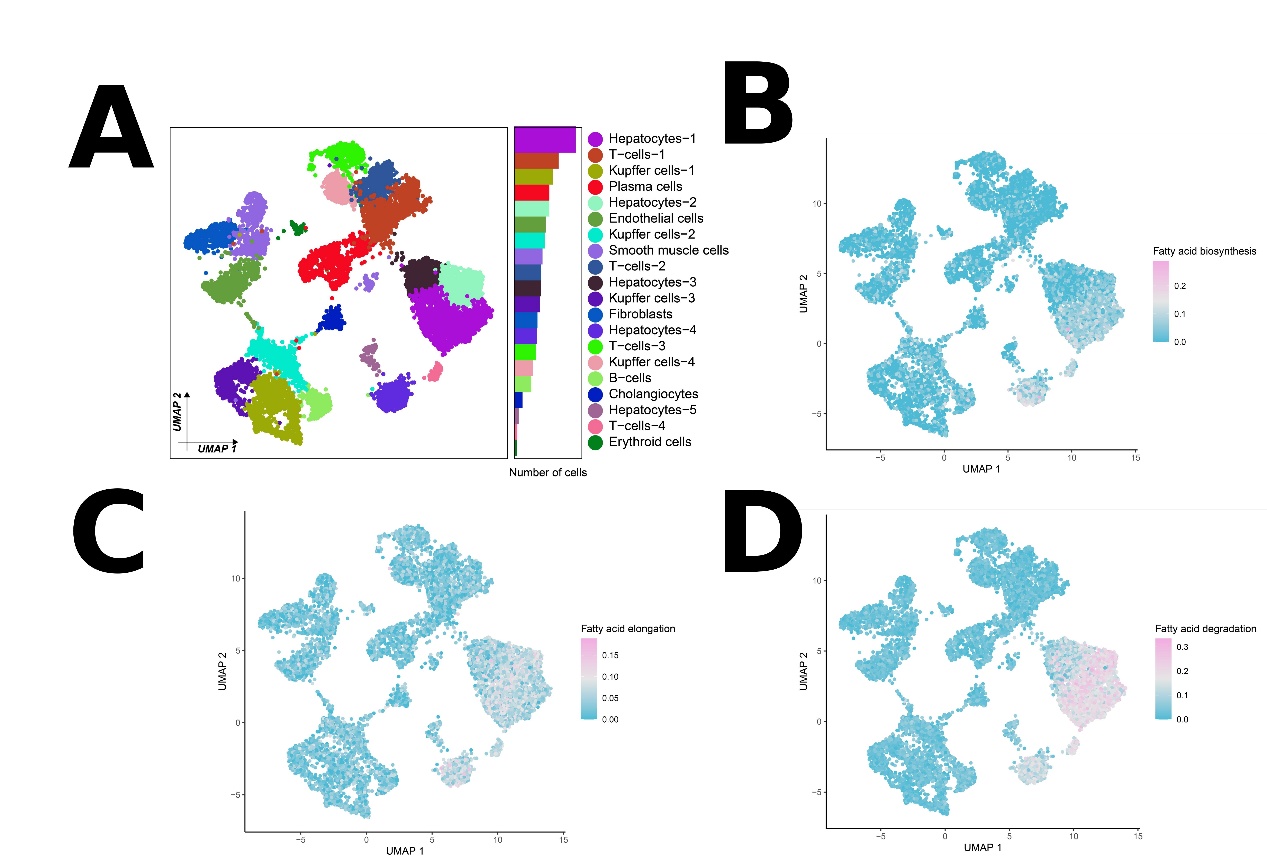
**

Supplementary Figure 2: Enrichment of Lipid Metabolism Pathways in Different Hepatic Cell Subpopulations from the HPA Database (A) UMAP plot depicting the clustering of different hepatic cell subpopulations based on single-cell RNA-seq data from human liver, with a bar chart indicating the number of cells in each subpopulation. The distinct clusters represent various hepatic cell types, including hepatocytes, T-cells, Kupffer cells, and others.(B) UMAP plot illustrating the enrichment of the fatty acid biosynthesis pathway across different hepatic cell subpopulations, highlighting specific clusters with elevated pathway activity.(C) UMAP plot showing the enrichment of the fatty acid elongation pathway within various hepatic cell subpopulations, indicating the differential involvement of this pathway among different cell types.D) UMAP plot displaying the enrichment of the fatty acid degradation pathway in different hepatic cell subpopulations, with certain clusters exhibiting higher activity levels.


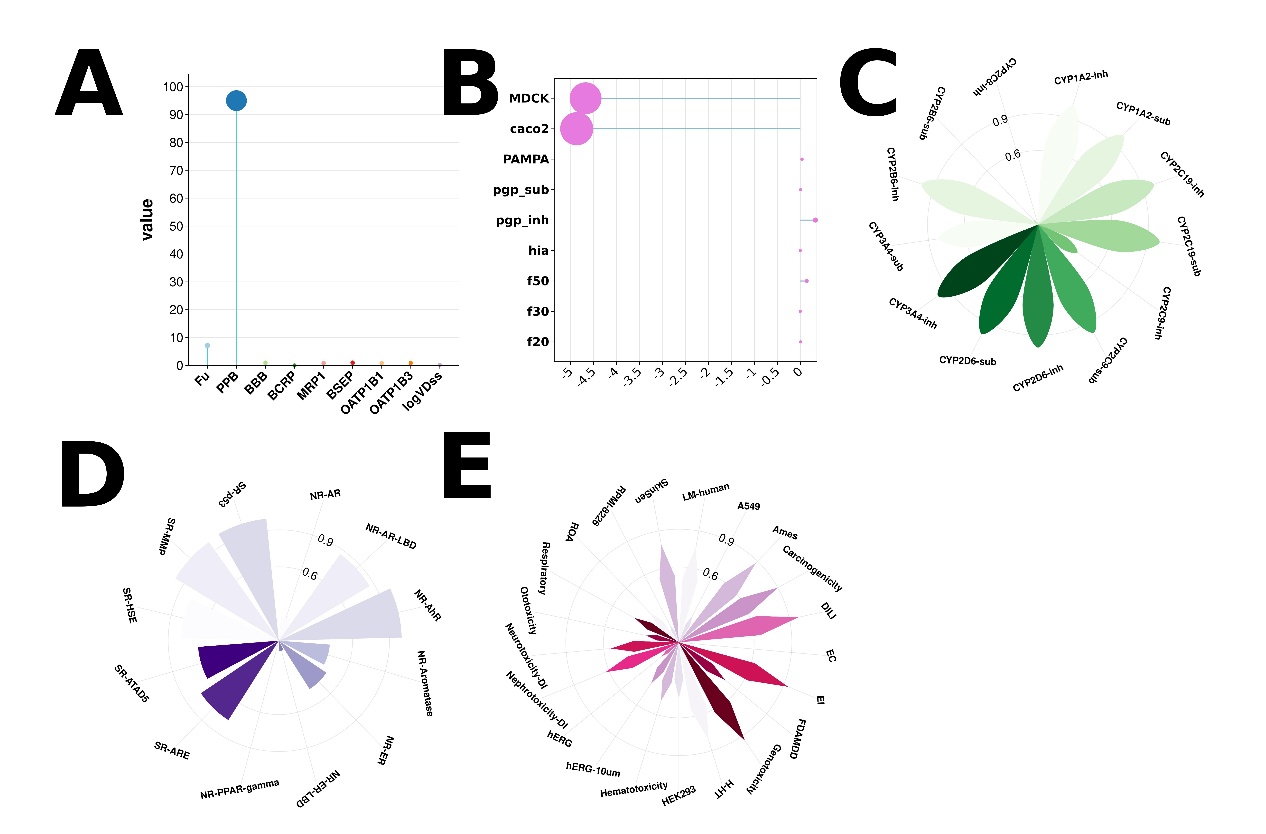


Supplementary Figure3:ADMET Analysis and Toxicity Profiling of Sesamin (SES)(A) Physicochemical properties and drug-likeness parameters of SES. The chart displays values for various properties including Fu (fraction unbound), PPB (plasma protein binding), BBB (blood-brain barrier permeability), BCBP (breast cancer resistance protein), MRP1 (multidrug resistance-associated protein 1), BSEP (bile salt export pump), OATP1B1 and OATP1B3 (organic anion transporting polypeptides), and logD (distribution coefficient).(B) Absorption profile of SES across different cell models. The chart shows permeability coefficients (in log scale) for MDCK (Madin-Darby Canine Kidney) cells, Caco-2 cells, PAMPA (Parallel Artificial Membrane Permeability Assay), Pgp substrate and inhibition potential, HIA (Human Intestinal Absorption), and F20, F30, F50 (oral bioavailability at 20, 30, and 50 mg/kg doses).(C) Metabolism profile of SES, illustrating its interaction with various cytochrome P450 enzymes. The radar chart displays substrate and inhibition potentials for CYP1A2, CYP2C9, CYP2C19, CYP2D6, CYP3A4, and other CYP isoforms.(D) Nuclear receptor interactions of SES. The radar chart shows the potential interactions of SES with various nuclear receptors including PXR, CAR, AhR, and others, which play crucial roles in xenobiotic metabolism and toxicity.(E) Toxicity profile of SES based on various in silico and in vitro assays. The radar chart displays predicted outcomes for different toxicity endpoints including carcinogenicity, mutagenicity, hepatotoxicity, cardiotoxicity (hERG inhibition), and specific cell line toxicities (A549, HepG2, etc.).


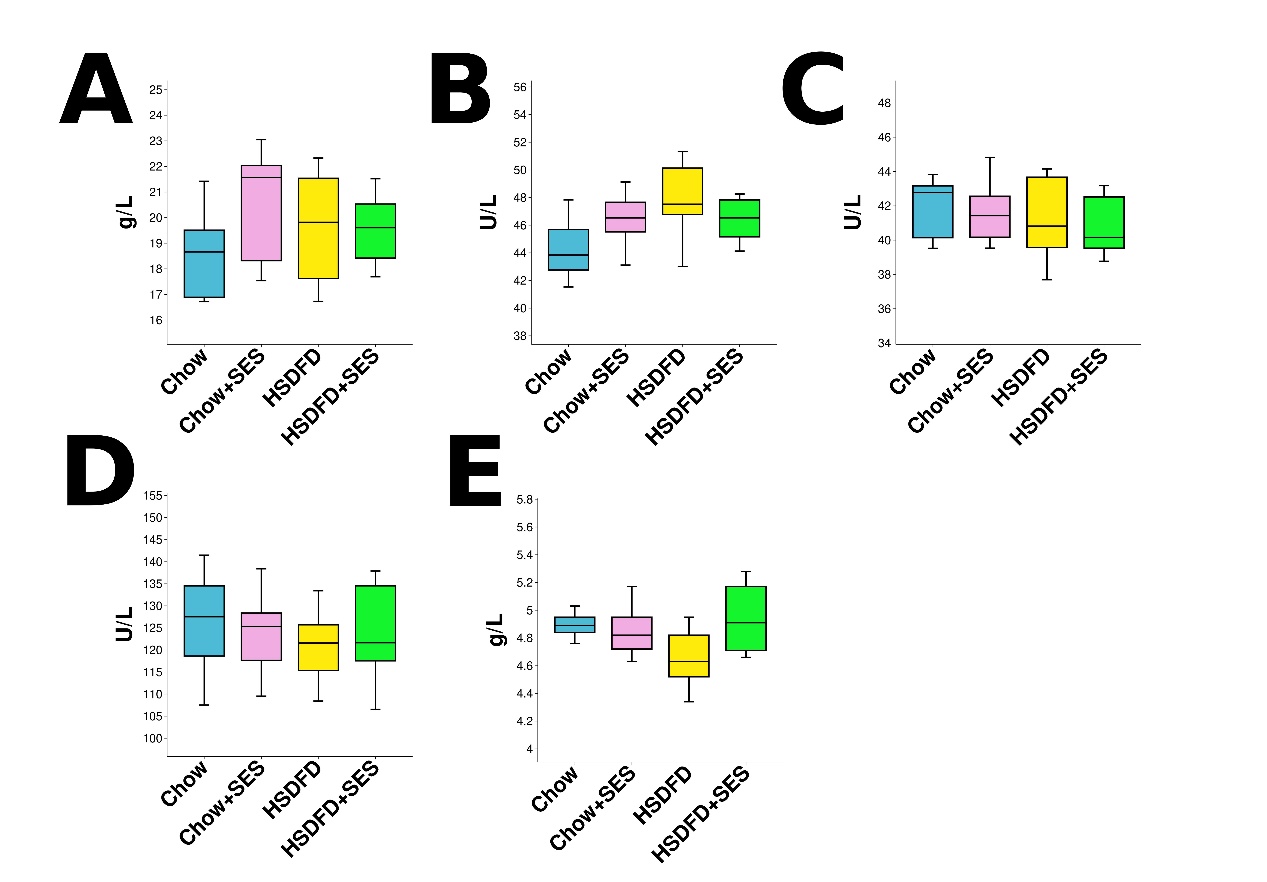


Supplementary Figure4:Changes in Liver Function Parameters in Mice Under Different Dietary Conditions (A) Serum albumin (ALB) levels (g/L) across different dietary groups.(B) Serum alkaline phosphatase (ALP) activity (U/L) across different dietary groups.(C) Serum alanine aminotransferase (ALT) activity (U/L) across different dietary groups.(D) Serum aspartate aminotransferase (AST) activity (U/L) across different dietary groups.(E) Serum total protein (TP) levels (g/L) across different dietary groups.


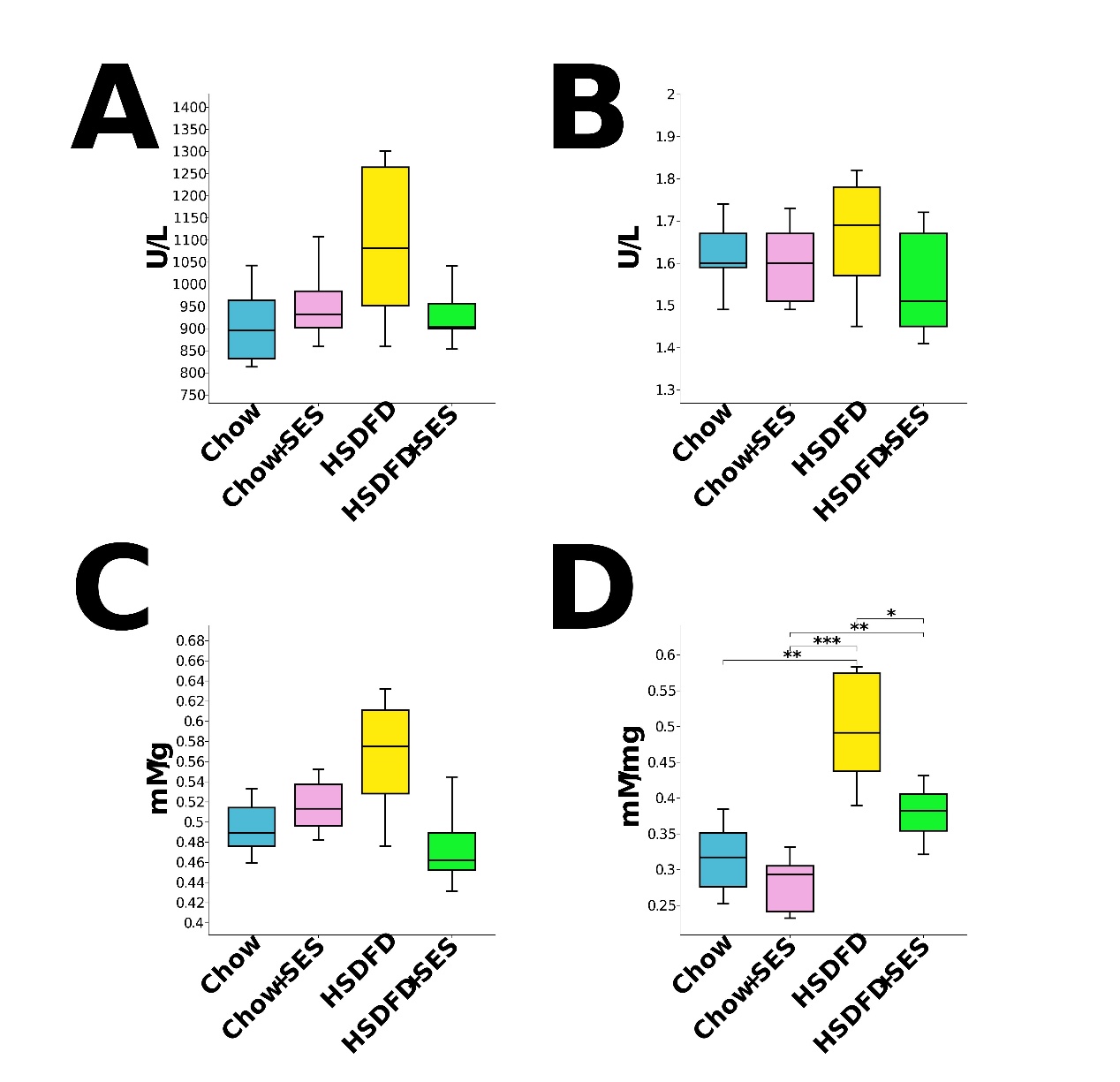


Supplementary Figure5:Changes in Hepatic Oxidative Stress Parameters in Mice Under Different Dietary Conditions (A) Glutathione peroxidase (GPX) activity (U/L) in liver tissue across different dietary groups.(B) Superoxide dismutase (SOD) activity (U/L) in liver tissue across different dietary groups.(C) Total antioxidant capacity (T-AOC) (mM/g) in liver tissue across different dietary groups.(D) Malondialdehyde (MDA) levels (mM/mg), a marker of lipid peroxidation, in liver tissue across different dietary groups.


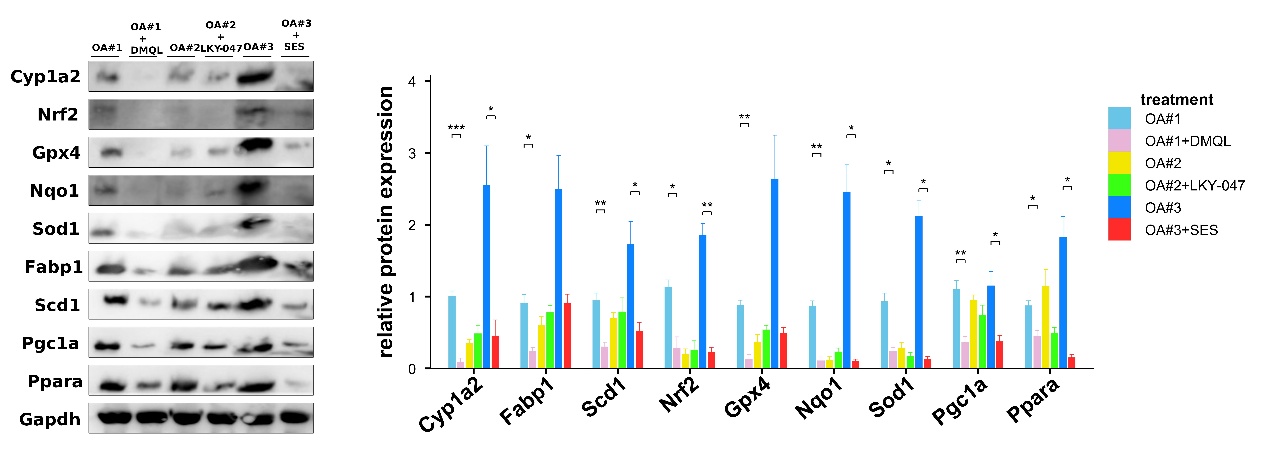


Supplementary Figure 6:Effects of sesamin, the CYP1A2 inhibitor 2,6-Dimethylquinoline(DMQL), and the negative control compound LKY-047 on CYP1A2, antioxidant stress response-related proteins, and lipid metabolism-related proteins in OA-treated AML12 cells.


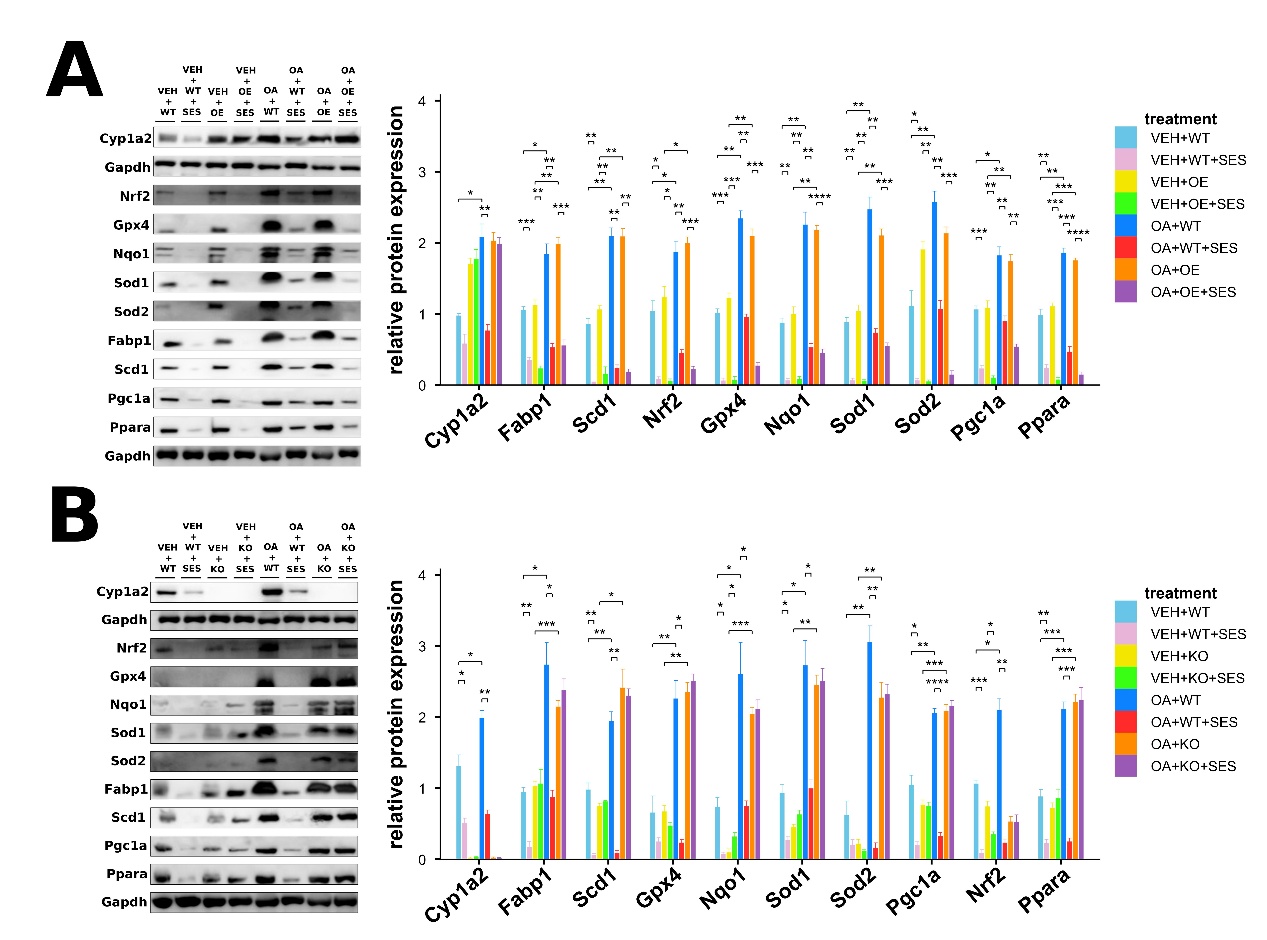


Supplementary Figure 7:Effects of CYP1A2 overexpression or knockout on oxidative stress response- and lipid metabolism-related proteins in AML12 hepatocytes.(A) Representative western blot images and quantitative analysis of Cyp1a2, oxidative stress response-related proteins (Nrf2, Gpx4, Nqo1, Sod1, and Sod2), and lipid metabolism-related proteins (Fabp1, Scd1, Pgc1α, and Pparα) in AML12 cells with Cyp1a2 overexpression under the indicated treatment conditions. Gapdh was used as the loading control.(B) Representative western blot images and quantitative analysis of Cyp1a2, oxidative stress response-related proteins (Nrf2, Gpx4, Nqo1, Sod1, and Sod2), and lipid metabolism-related proteins (Fabp1, Scd1, Pgc1α, and Pparα) in AML12 cells with Cyp1a2 knockout under the indicated treatment conditions. Gapdh was used as the loading control.


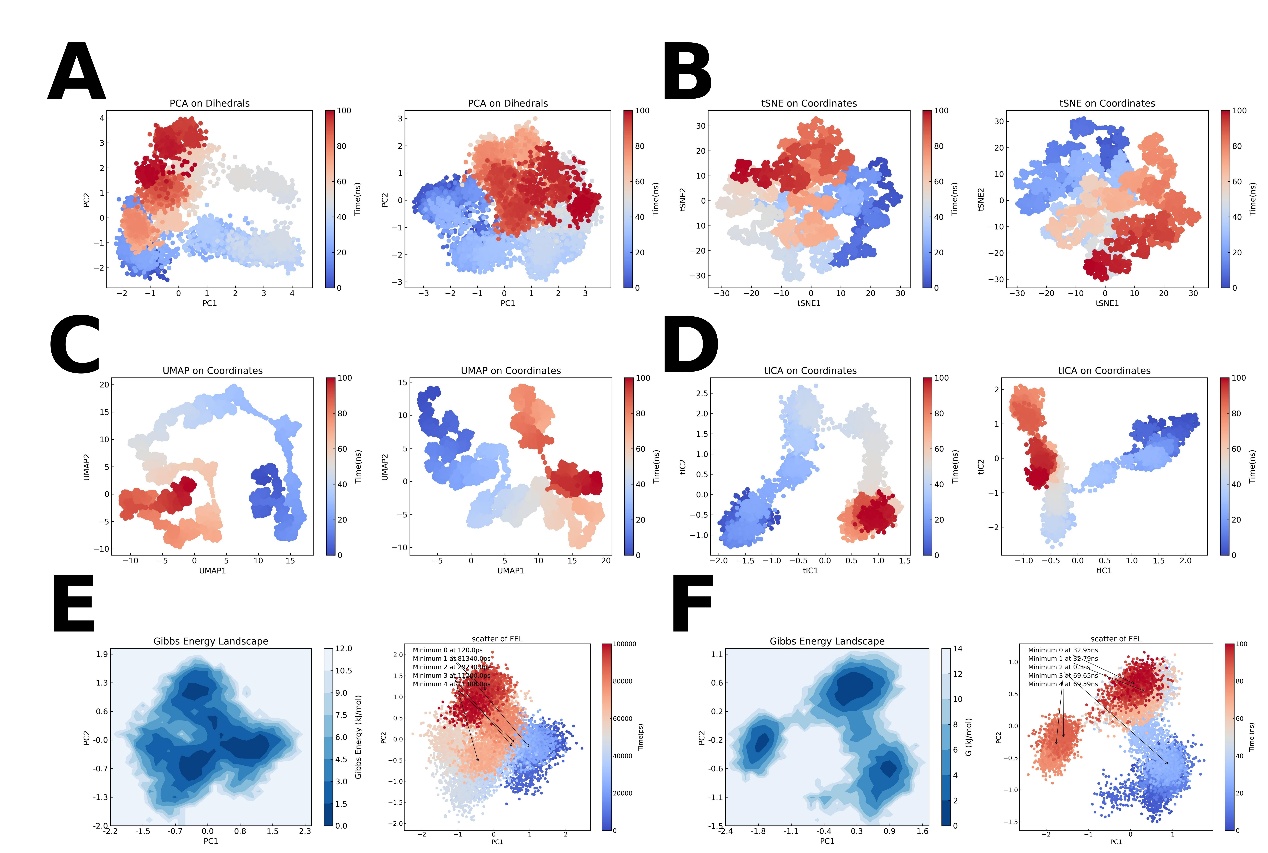


Supplementary Figure8:Conformational Analysis and Energy Landscape of CYP1A2 in Apo and Sesamin-Bound States (A) Principal Component Analysis (PCA) based on dihedral angles of CYP1A2. Left: Apo state. Right: Sesamin (SES)-bound state. The color gradient represents simulation time progression.(B) t-SNE (t-Distributed Stochastic Neighbor Embedding) analysis based on atomic coordinates of CYP1A2. Left: Apo state. Right: SES-bound state. The color gradient indicates simulation time.(C) UMAP (Uniform Manifold Approximation and Projection) analysis based on atomic coordinates of CYP1A2. Left: Apo state. Right: SES-bound state. The color gradient shows simulation time progression.(D) tICA (time-lagged Independent Component Analysis) based on atomic coordinates of CYP1A2. Left: Apo state. Right: SES-bound state. The color gradient represents simulation time.(E) Free Energy Landscape (FEL) of CYP1A2 in the Apo state. Left: Gibbs energy landscape plotted against the first two principal components. Right: Scatter plot showing the time required to reach energy minima (conformational traps). Color gradient indicates simulation time.(F) Free Energy Landscape (FEL) of CYP1A2 in the SES-bound state. Left: Gibbs energy landscape plotted against the first two principal components. Right: Scatter plot showing the time required to reach energy minima. Color gradient represents simulation time.


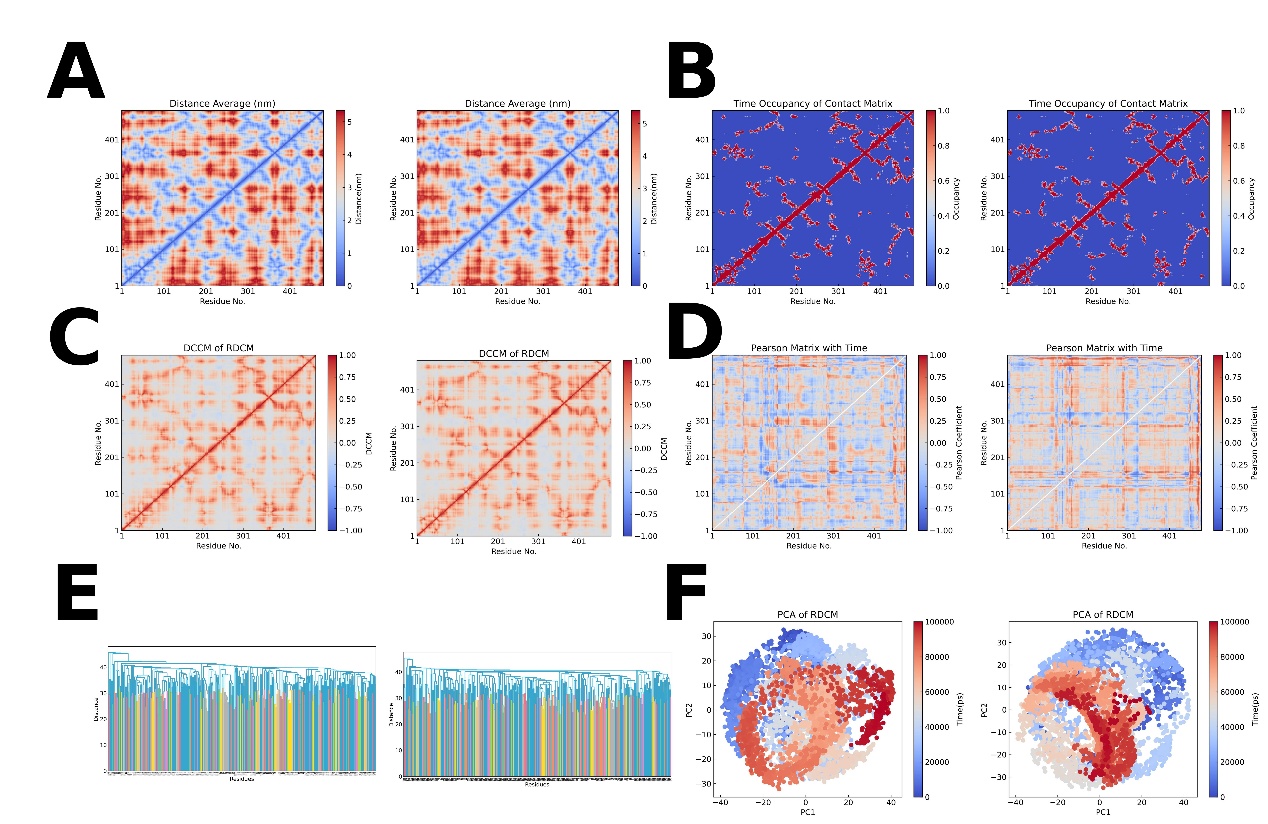


Supplementary Figure9: Residue Distance-Based Correlation Analysis of CYP1A2 in Apo and Sesamin-Bound States(A) Average distance matrices between residues. Left: Apo state. Right: Sesamin (SES)-bound state. Color scale indicates distance in nm.(B) Time occupancy of contact matrices. Left: Apo state. Right: SES-bound state. Color scale represents the fraction of simulation time that residues are in contact.(C) Dynamic Cross-Correlation Matrices (DCCM) based on Residue Distance Correlation Matrices (RDCM). Left: Apo state. Right: SES-bound state. Color scale indicates the degree of correlated motion between residues.(D) Pearson correlation matrices of residue distances with simulation time. Left: Apo state. Right: SES-bound state. Color scale shows the strength and direction of correlation.(E) Hierarchical clustering dendrograms based on residue distances. Left: Apo state. Right: SES-bound state. Colors represent different clusters of residues with similar distance patterns.(F) Principal Component Analysis (PCA) of Residue Distance Correlation Matrices (RDCM). Left: Apo state. Right: SES-bound state. Color gradient indicates simulation time progression.


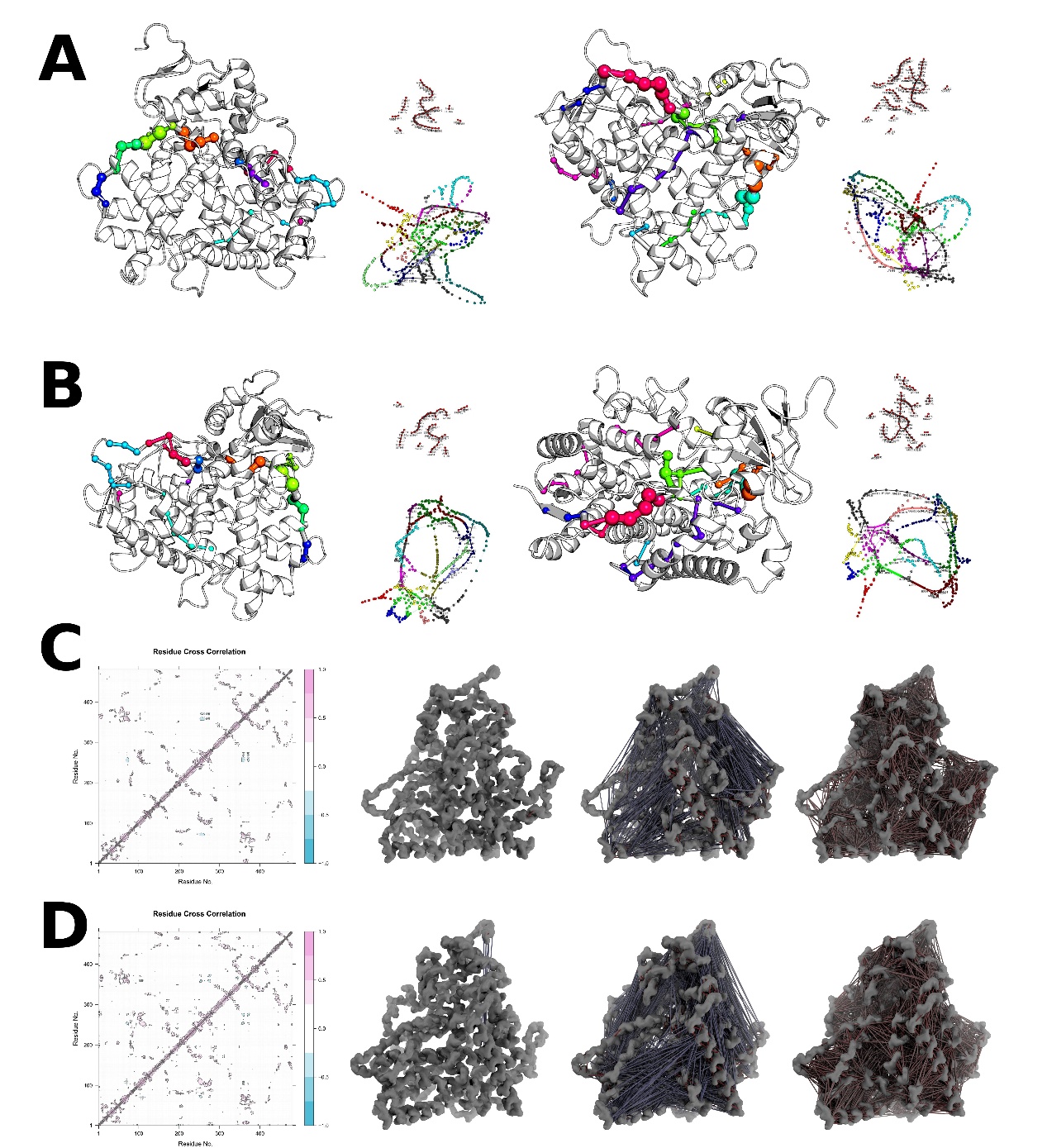


Supplementary Figure 10:Structural and Dynamic Analysis of CYP1A2 in Apo and Sesamin-Bound States (A) Residue shortest path networks of CYP1A2 in apo (left) and sesamin-bound (right) states. The protein structure is shown in grey ribbon representation, with colored spheres and lines representing key residues and their connections in the shortest path network. Adjacent to each protein structure is the corresponding network diagram. (B) Residue second shortest path networks of CYP1A2 in apo (left) and sesamin-bound (right) states. Similar to panel A, but showing alternative communication pathways within the protein. (C) Dynamic Cross-Correlation Matrix (DCCM) analysis of CYP1A2 in the apo state. Left: DCCM plot showing correlations between residue motions. Right: Three-dimensional representations of the protein showing correlated motions at different hierarchical levels, from local (left) to global (right) correlations.(D) DCCM analysis of CYP1A2 in the sesamin-bound state. Arranged similarly to panel C, showing how sesamin binding affects residue motion correlations throughout the protein.


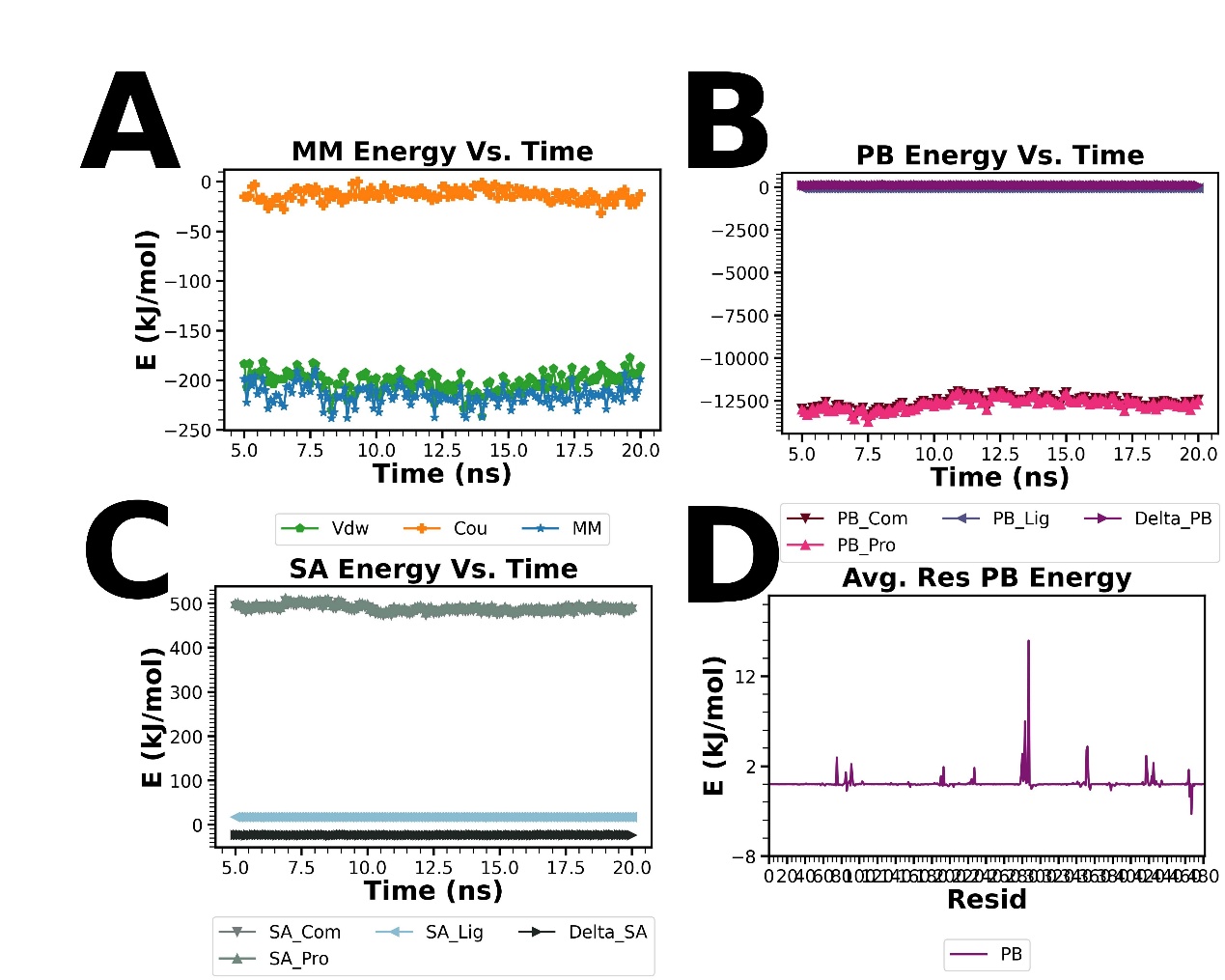


Supplementary Figure11:Molecular Dynamics Analysis of Sesamin (SES) Interaction with CYP1A2 (A) MM Energy vs. Time: Time evolution of van der Waals (Vdw), Coulombic (Cou), and total Molecular Mechanics (MM) energy components during the simulation.(B) PB Energy vs. Time: Time evolution of Poisson-Boltzmann (PB) energy components, including complex (PB_Com), ligand (PB_Lig), protein (PB_Pro), and total PB energy (Delta_PB).(C) SA Energy vs. Time: Time evolution of Surface Area (SA) energy components, including complex (SA_Com), ligand (SA_Lig), protein (SA_Pro), and total SA energy (Delta_SA).(D) Avg. Res PB Energy: Residue-wise decomposition of the average PB energy contribution.
