# Supplementary figures and images for: Sesamin Ameliorates High‐Sugar, High‐Fat Diet‐Induced Hepatic Dysfunction via CYP1A2‐Mediated Regulation of Lipid Metabolism and Oxidative Stress
Source: J Cell Mol Med. 2026 Jul 17;30(14):e71274. doi: 10.1111/jcmm.71274 (PMC13378100; doi:10.1111/jcmm.71274)

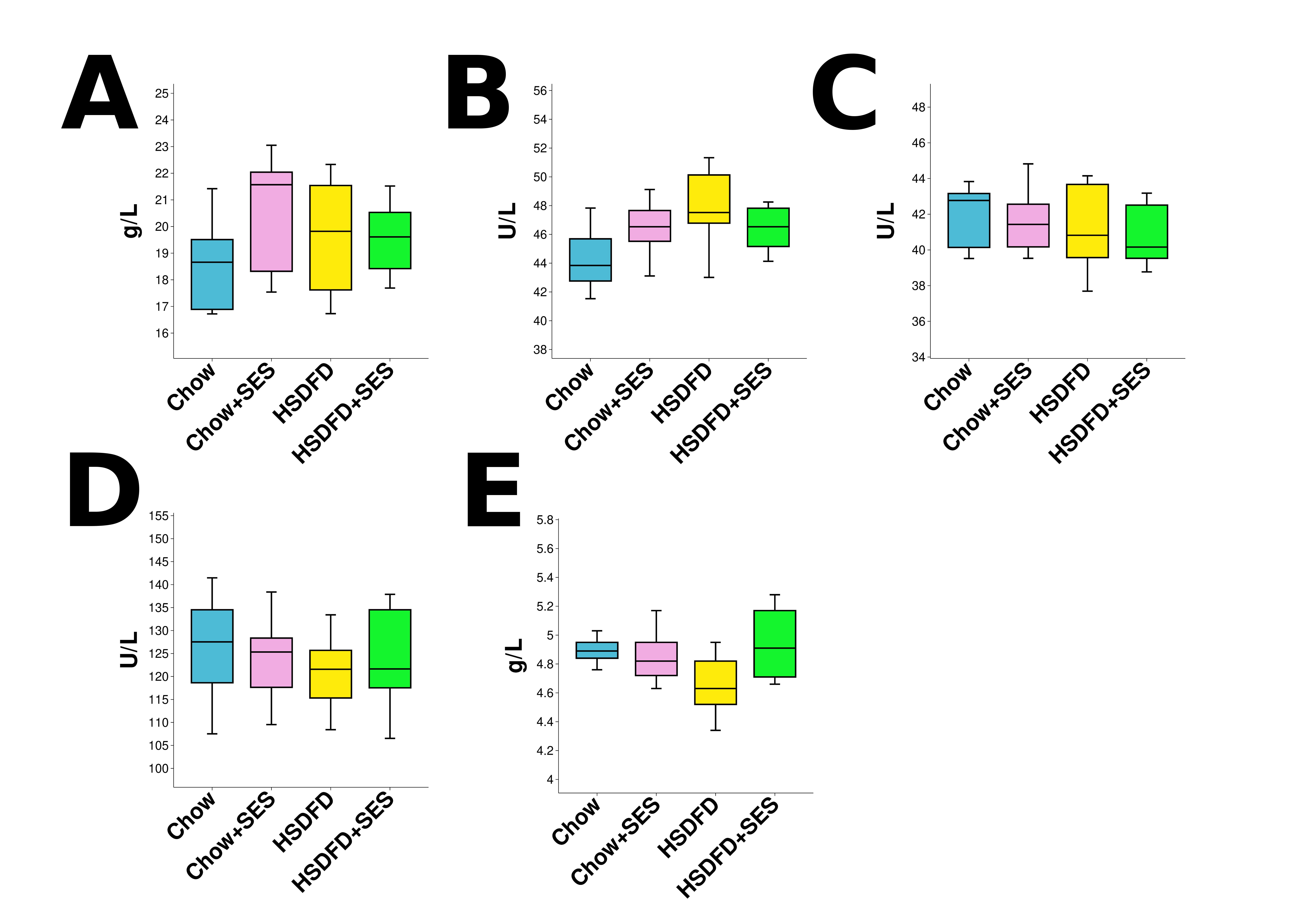

Supplement: Supplementary file 1 — Figure S1: Single‐cell analysis of hepatocyte subpopulations under chow and high‐sugar, high‐fat diet (HSHFD) conditions. (A) Metabolic activity scores across different hepatocyte subpopulations. (B) Heatmap showing activity of various metabolic pathways in different hepatocyte subpopulations. (C) UMAP plots illustrating fatty acid elongation activity in hepatocyte subpopulations under chow and HSHFD conditions. (D) UMAP plots showing fatty acid biosynthesis activity in hepatocyte subpopulations under chow and HSHFD conditions. (E) UMAP plots depicting fatty acid degradation activity in hepatocyte subpopulations under chow and HSHFD conditions. (F) UMAP plots showing activity of xenobiotic metabolism by cytochrome P450 in hepatocyte subpopulations under chow and HSHFD conditions. (G) Mapping of hepatocyte subpopulations from chow and HSHFD conditions to human liver single‐cell atlas. (H) Proportion of hepatocyte subpopulations mapped to different human hepatocyte subtypes under chow and HSHFD conditions. (I) UMAP plots showing how different mouse hepatocyte subpopulations map to human liver atlas hepatocyte subtypes. Figure S2: Enrichment of Lipid Metabolism Pathways in Different Hepatic Cell Subpopulations from the HPA Database (A) UMAP plot depicting the clustering of different hepatic cell subpopulations based on single‐cell RNA‐seq data from human liver, with a bar chart indicating the number of cells in each subpopulation. The distinct clusters represent various hepatic cell types, including hepatocytes, T‐cells, Kupffer cells, and others. (B) UMAP plot illustrating the enrichment of the fatty acid biosynthesis pathway across different hepatic cell subpopulations, highlighting specific clusters with elevated pathway activity. (C) UMAP plot showing the enrichment of the fatty acid elongation pathway within various hepatic cell subpopulations, indicating the differential involvement of this pathway among different cell types. (D) UMAP plot displaying the enrichme [file JCMM-30-e71274-s001.zip › jcmm71274-sup-0004-FigureS4@supplementaryfigure4.jpg]

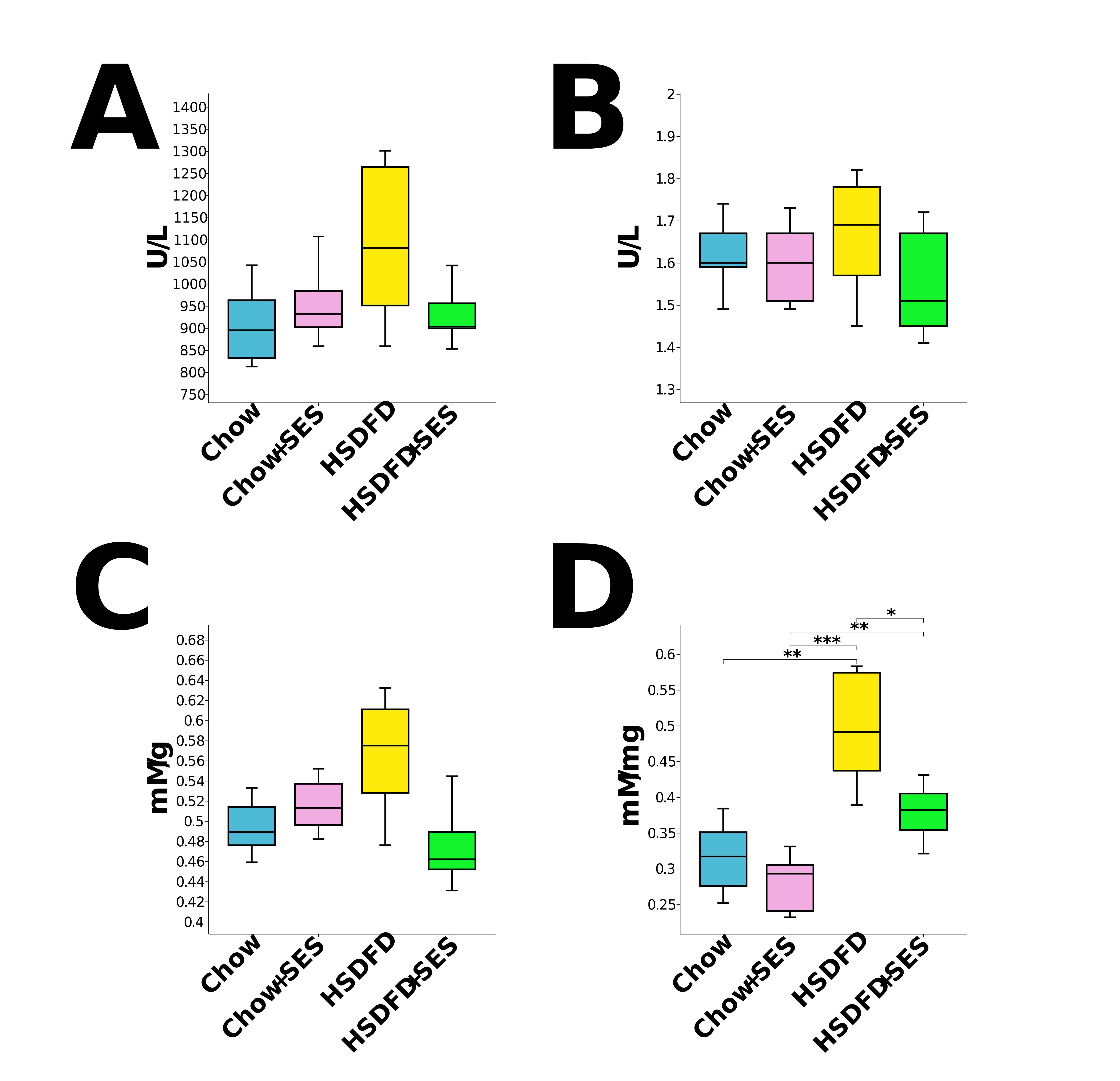

Supplement: Supplementary file 1 — Figure S1: Single‐cell analysis of hepatocyte subpopulations under chow and high‐sugar, high‐fat diet (HSHFD) conditions. (A) Metabolic activity scores across different hepatocyte subpopulations. (B) Heatmap showing activity of various metabolic pathways in different hepatocyte subpopulations. (C) UMAP plots illustrating fatty acid elongation activity in hepatocyte subpopulations under chow and HSHFD conditions. (D) UMAP plots showing fatty acid biosynthesis activity in hepatocyte subpopulations under chow and HSHFD conditions. (E) UMAP plots depicting fatty acid degradation activity in hepatocyte subpopulations under chow and HSHFD conditions. (F) UMAP plots showing activity of xenobiotic metabolism by cytochrome P450 in hepatocyte subpopulations under chow and HSHFD conditions. (G) Mapping of hepatocyte subpopulations from chow and HSHFD conditions to human liver single‐cell atlas. (H) Proportion of hepatocyte subpopulations mapped to different human hepatocyte subtypes under chow and HSHFD conditions. (I) UMAP plots showing how different mouse hepatocyte subpopulations map to human liver atlas hepatocyte subtypes. Figure S2: Enrichment of Lipid Metabolism Pathways in Different Hepatic Cell Subpopulations from the HPA Database (A) UMAP plot depicting the clustering of different hepatic cell subpopulations based on single‐cell RNA‐seq data from human liver, with a bar chart indicating the number of cells in each subpopulation. The distinct clusters represent various hepatic cell types, including hepatocytes, T‐cells, Kupffer cells, and others. (B) UMAP plot illustrating the enrichment of the fatty acid biosynthesis pathway across different hepatic cell subpopulations, highlighting specific clusters with elevated pathway activity. (C) UMAP plot showing the enrichment of the fatty acid elongation pathway within various hepatic cell subpopulations, indicating the differential involvement of this pathway among different cell types. (D) UMAP plot displaying the enrichme [file JCMM-30-e71274-s001.zip › jcmm71274-sup-0005-FigureS5@supplementaryfigure5.jpg]

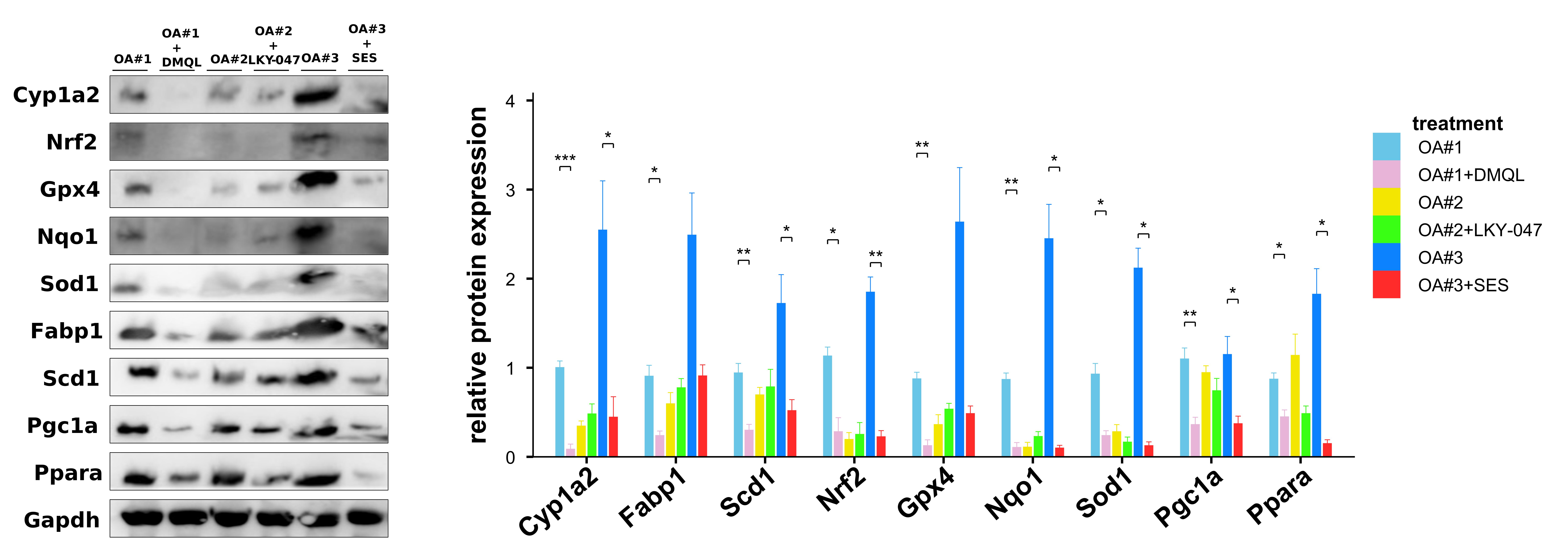

Supplement: Supplementary file 1 — Figure S1: Single‐cell analysis of hepatocyte subpopulations under chow and high‐sugar, high‐fat diet (HSHFD) conditions. (A) Metabolic activity scores across different hepatocyte subpopulations. (B) Heatmap showing activity of various metabolic pathways in different hepatocyte subpopulations. (C) UMAP plots illustrating fatty acid elongation activity in hepatocyte subpopulations under chow and HSHFD conditions. (D) UMAP plots showing fatty acid biosynthesis activity in hepatocyte subpopulations under chow and HSHFD conditions. (E) UMAP plots depicting fatty acid degradation activity in hepatocyte subpopulations under chow and HSHFD conditions. (F) UMAP plots showing activity of xenobiotic metabolism by cytochrome P450 in hepatocyte subpopulations under chow and HSHFD conditions. (G) Mapping of hepatocyte subpopulations from chow and HSHFD conditions to human liver single‐cell atlas. (H) Proportion of hepatocyte subpopulations mapped to different human hepatocyte subtypes under chow and HSHFD conditions. (I) UMAP plots showing how different mouse hepatocyte subpopulations map to human liver atlas hepatocyte subtypes. Figure S2: Enrichment of Lipid Metabolism Pathways in Different Hepatic Cell Subpopulations from the HPA Database (A) UMAP plot depicting the clustering of different hepatic cell subpopulations based on single‐cell RNA‐seq data from human liver, with a bar chart indicating the number of cells in each subpopulation. The distinct clusters represent various hepatic cell types, including hepatocytes, T‐cells, Kupffer cells, and others. (B) UMAP plot illustrating the enrichment of the fatty acid biosynthesis pathway across different hepatic cell subpopulations, highlighting specific clusters with elevated pathway activity. (C) UMAP plot showing the enrichment of the fatty acid elongation pathway within various hepatic cell subpopulations, indicating the differential involvement of this pathway among different cell types. (D) UMAP plot displaying the enrichme [file JCMM-30-e71274-s001.zip › jcmm71274-sup-0006-FigureS6@supplementaryfigure6.jpg]

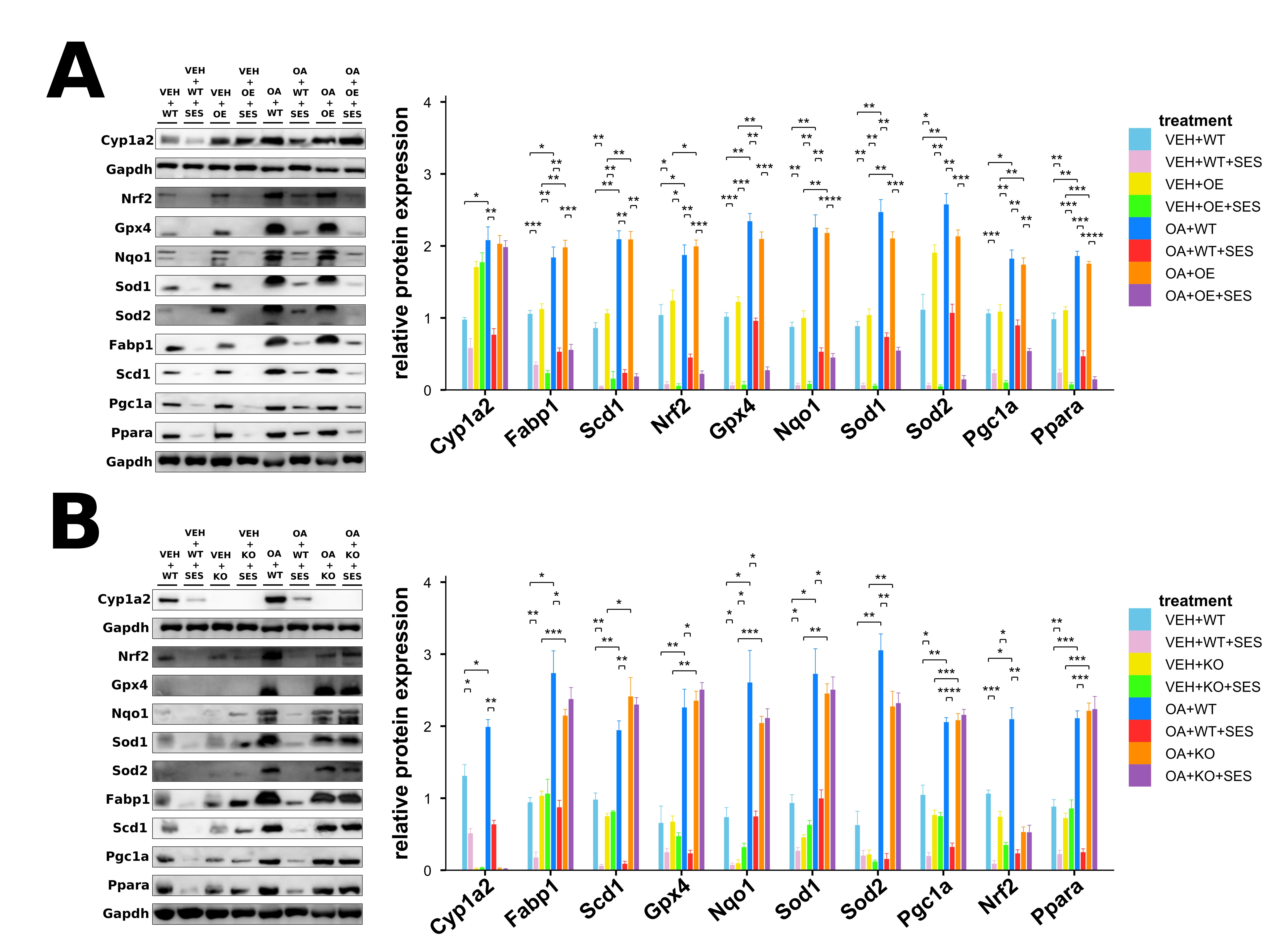

Supplement: Supplementary file 1 — Figure S1: Single‐cell analysis of hepatocyte subpopulations under chow and high‐sugar, high‐fat diet (HSHFD) conditions. (A) Metabolic activity scores across different hepatocyte subpopulations. (B) Heatmap showing activity of various metabolic pathways in different hepatocyte subpopulations. (C) UMAP plots illustrating fatty acid elongation activity in hepatocyte subpopulations under chow and HSHFD conditions. (D) UMAP plots showing fatty acid biosynthesis activity in hepatocyte subpopulations under chow and HSHFD conditions. (E) UMAP plots depicting fatty acid degradation activity in hepatocyte subpopulations under chow and HSHFD conditions. (F) UMAP plots showing activity of xenobiotic metabolism by cytochrome P450 in hepatocyte subpopulations under chow and HSHFD conditions. (G) Mapping of hepatocyte subpopulations from chow and HSHFD conditions to human liver single‐cell atlas. (H) Proportion of hepatocyte subpopulations mapped to different human hepatocyte subtypes under chow and HSHFD conditions. (I) UMAP plots showing how different mouse hepatocyte subpopulations map to human liver atlas hepatocyte subtypes. Figure S2: Enrichment of Lipid Metabolism Pathways in Different Hepatic Cell Subpopulations from the HPA Database (A) UMAP plot depicting the clustering of different hepatic cell subpopulations based on single‐cell RNA‐seq data from human liver, with a bar chart indicating the number of cells in each subpopulation. The distinct clusters represent various hepatic cell types, including hepatocytes, T‐cells, Kupffer cells, and others. (B) UMAP plot illustrating the enrichment of the fatty acid biosynthesis pathway across different hepatic cell subpopulations, highlighting specific clusters with elevated pathway activity. (C) UMAP plot showing the enrichment of the fatty acid elongation pathway within various hepatic cell subpopulations, indicating the differential involvement of this pathway among different cell types. (D) UMAP plot displaying the enrichme [file JCMM-30-e71274-s001.zip › jcmm71274-sup-0007-FigureS7@supplementaryfigure7.jpg]

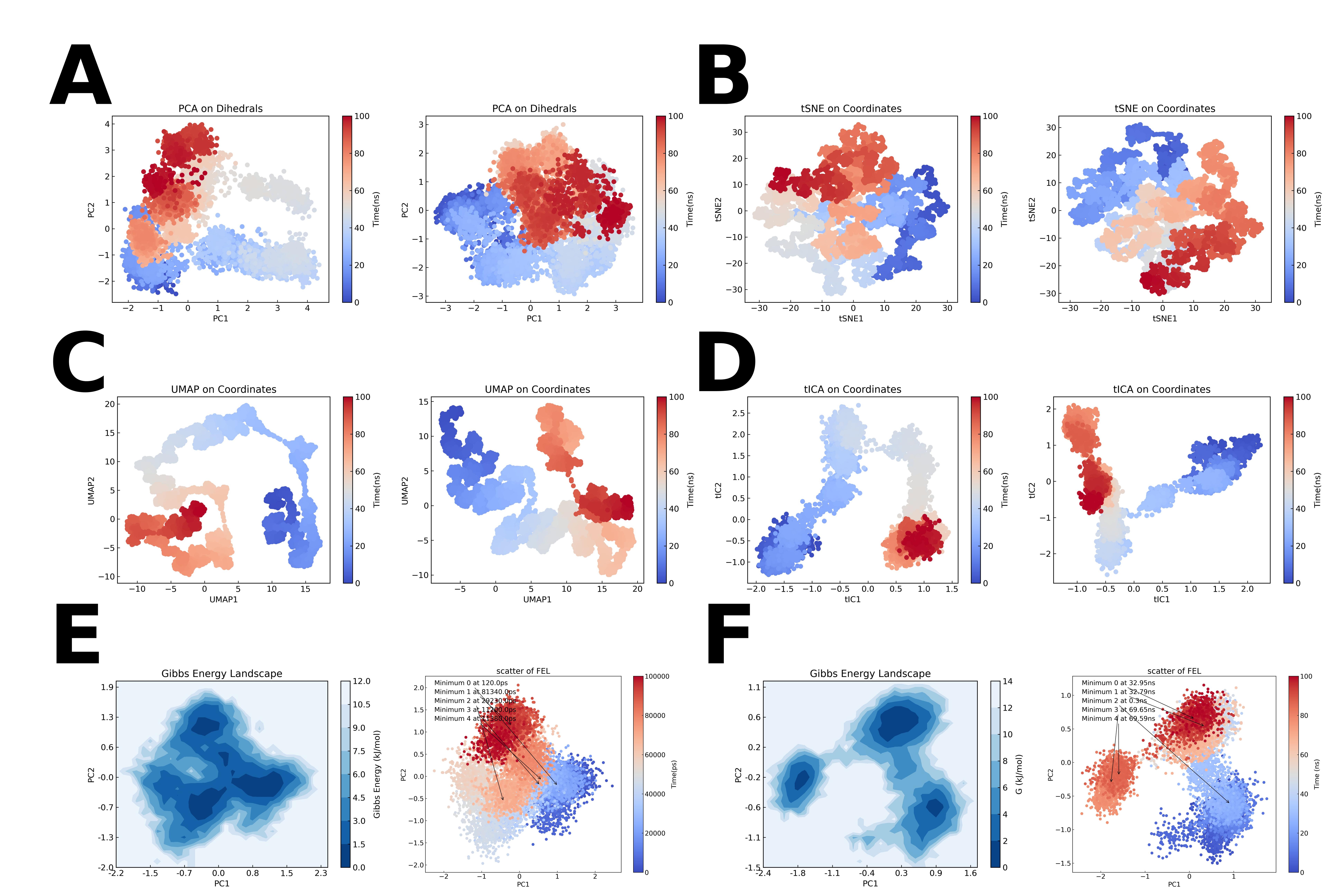

Supplement: Supplementary file 1 — Figure S1: Single‐cell analysis of hepatocyte subpopulations under chow and high‐sugar, high‐fat diet (HSHFD) conditions. (A) Metabolic activity scores across different hepatocyte subpopulations. (B) Heatmap showing activity of various metabolic pathways in different hepatocyte subpopulations. (C) UMAP plots illustrating fatty acid elongation activity in hepatocyte subpopulations under chow and HSHFD conditions. (D) UMAP plots showing fatty acid biosynthesis activity in hepatocyte subpopulations under chow and HSHFD conditions. (E) UMAP plots depicting fatty acid degradation activity in hepatocyte subpopulations under chow and HSHFD conditions. (F) UMAP plots showing activity of xenobiotic metabolism by cytochrome P450 in hepatocyte subpopulations under chow and HSHFD conditions. (G) Mapping of hepatocyte subpopulations from chow and HSHFD conditions to human liver single‐cell atlas. (H) Proportion of hepatocyte subpopulations mapped to different human hepatocyte subtypes under chow and HSHFD conditions. (I) UMAP plots showing how different mouse hepatocyte subpopulations map to human liver atlas hepatocyte subtypes. Figure S2: Enrichment of Lipid Metabolism Pathways in Different Hepatic Cell Subpopulations from the HPA Database (A) UMAP plot depicting the clustering of different hepatic cell subpopulations based on single‐cell RNA‐seq data from human liver, with a bar chart indicating the number of cells in each subpopulation. The distinct clusters represent various hepatic cell types, including hepatocytes, T‐cells, Kupffer cells, and others. (B) UMAP plot illustrating the enrichment of the fatty acid biosynthesis pathway across different hepatic cell subpopulations, highlighting specific clusters with elevated pathway activity. (C) UMAP plot showing the enrichment of the fatty acid elongation pathway within various hepatic cell subpopulations, indicating the differential involvement of this pathway among different cell types. (D) UMAP plot displaying the enrichme [file JCMM-30-e71274-s001.zip › jcmm71274-sup-0008-FigureS8@supplementaryfigure8.jpg]

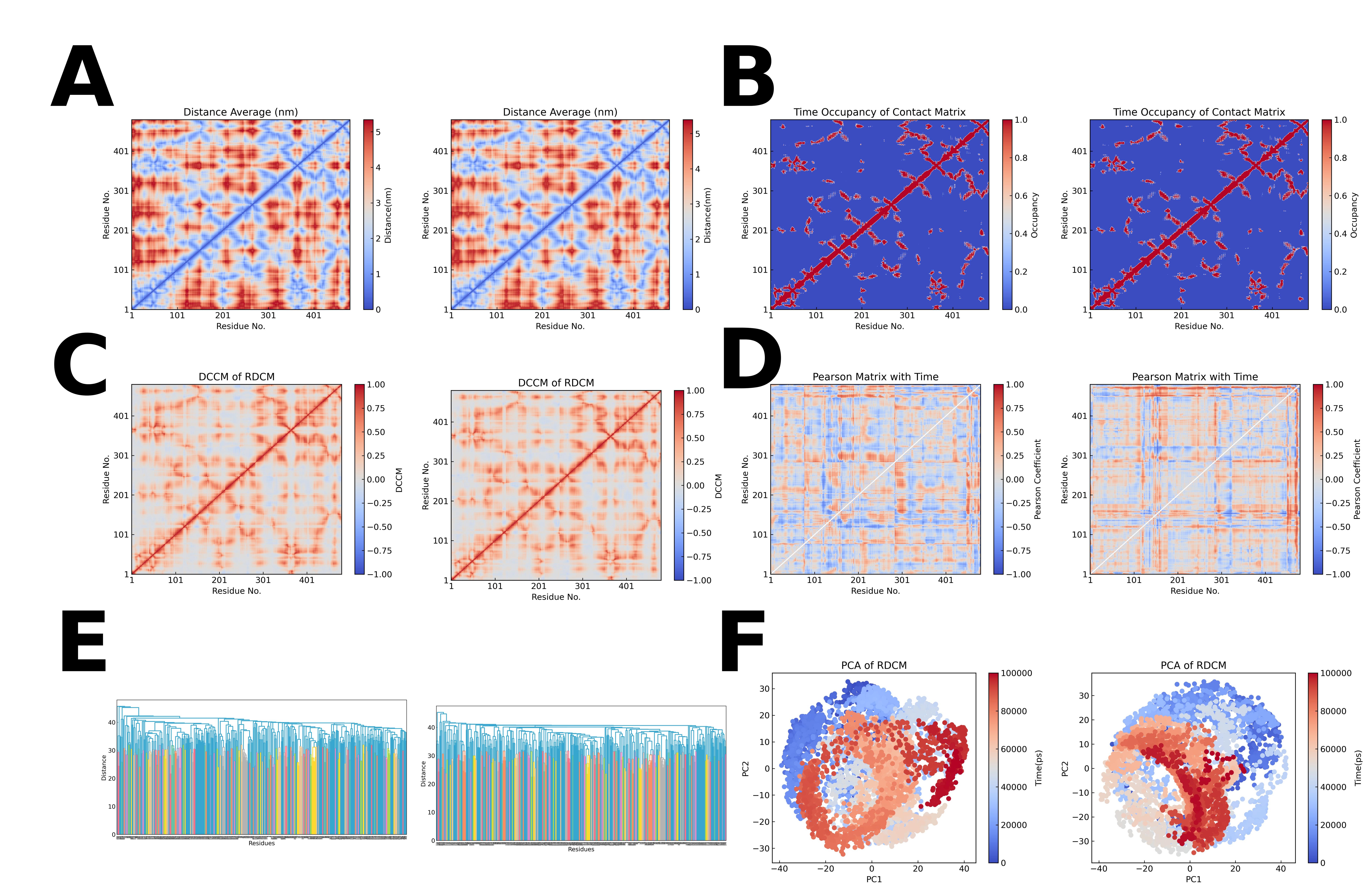

Supplement: Supplementary file 1 — Figure S1: Single‐cell analysis of hepatocyte subpopulations under chow and high‐sugar, high‐fat diet (HSHFD) conditions. (A) Metabolic activity scores across different hepatocyte subpopulations. (B) Heatmap showing activity of various metabolic pathways in different hepatocyte subpopulations. (C) UMAP plots illustrating fatty acid elongation activity in hepatocyte subpopulations under chow and HSHFD conditions. (D) UMAP plots showing fatty acid biosynthesis activity in hepatocyte subpopulations under chow and HSHFD conditions. (E) UMAP plots depicting fatty acid degradation activity in hepatocyte subpopulations under chow and HSHFD conditions. (F) UMAP plots showing activity of xenobiotic metabolism by cytochrome P450 in hepatocyte subpopulations under chow and HSHFD conditions. (G) Mapping of hepatocyte subpopulations from chow and HSHFD conditions to human liver single‐cell atlas. (H) Proportion of hepatocyte subpopulations mapped to different human hepatocyte subtypes under chow and HSHFD conditions. (I) UMAP plots showing how different mouse hepatocyte subpopulations map to human liver atlas hepatocyte subtypes. Figure S2: Enrichment of Lipid Metabolism Pathways in Different Hepatic Cell Subpopulations from the HPA Database (A) UMAP plot depicting the clustering of different hepatic cell subpopulations based on single‐cell RNA‐seq data from human liver, with a bar chart indicating the number of cells in each subpopulation. The distinct clusters represent various hepatic cell types, including hepatocytes, T‐cells, Kupffer cells, and others. (B) UMAP plot illustrating the enrichment of the fatty acid biosynthesis pathway across different hepatic cell subpopulations, highlighting specific clusters with elevated pathway activity. (C) UMAP plot showing the enrichment of the fatty acid elongation pathway within various hepatic cell subpopulations, indicating the differential involvement of this pathway among different cell types. (D) UMAP plot displaying the enrichme [file JCMM-30-e71274-s001.zip › jcmm71274-sup-0009-FigureS9@supplementaryfigure9.jpg]

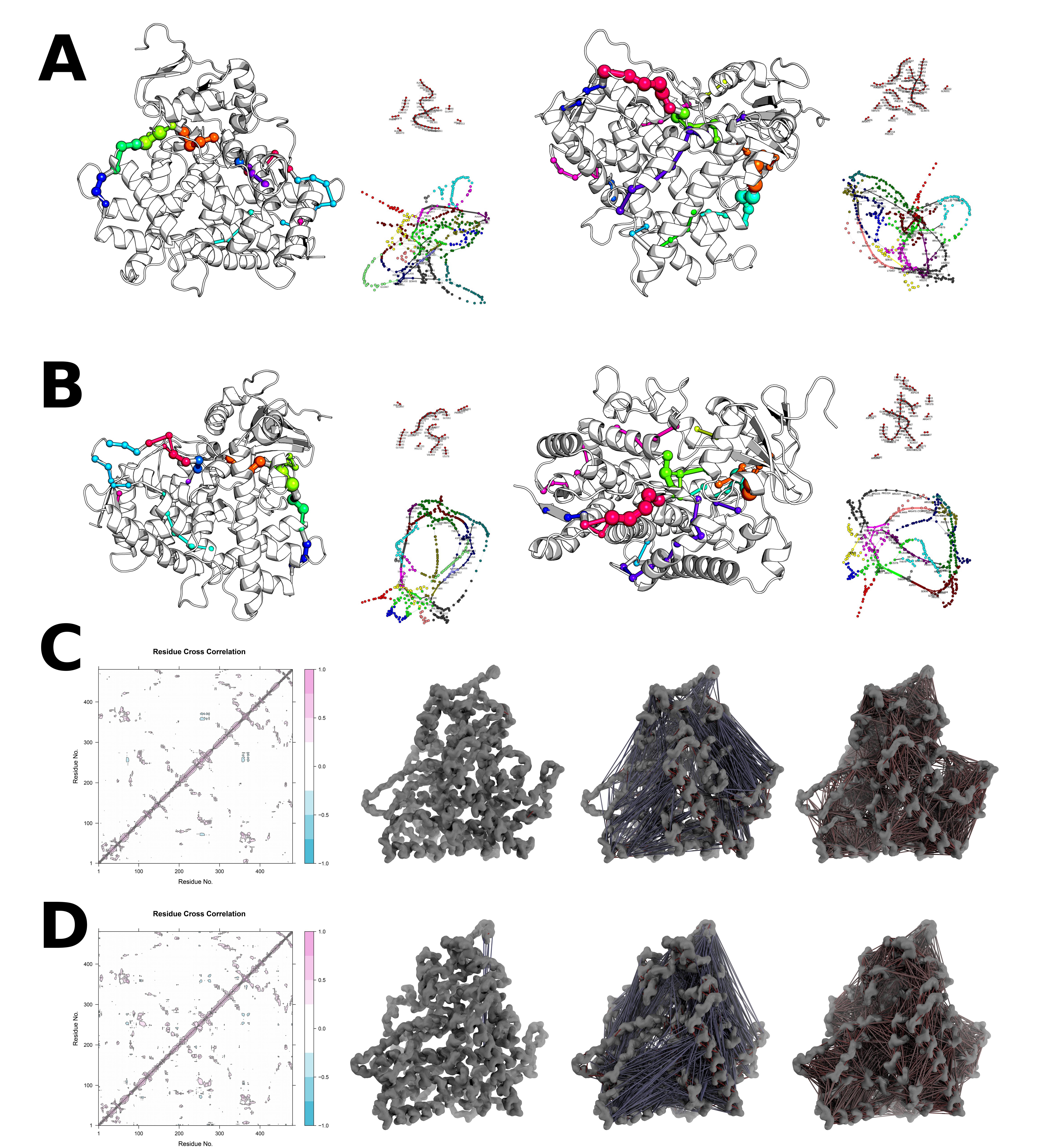

Supplement: Supplementary file 1 — Figure S1: Single‐cell analysis of hepatocyte subpopulations under chow and high‐sugar, high‐fat diet (HSHFD) conditions. (A) Metabolic activity scores across different hepatocyte subpopulations. (B) Heatmap showing activity of various metabolic pathways in different hepatocyte subpopulations. (C) UMAP plots illustrating fatty acid elongation activity in hepatocyte subpopulations under chow and HSHFD conditions. (D) UMAP plots showing fatty acid biosynthesis activity in hepatocyte subpopulations under chow and HSHFD conditions. (E) UMAP plots depicting fatty acid degradation activity in hepatocyte subpopulations under chow and HSHFD conditions. (F) UMAP plots showing activity of xenobiotic metabolism by cytochrome P450 in hepatocyte subpopulations under chow and HSHFD conditions. (G) Mapping of hepatocyte subpopulations from chow and HSHFD conditions to human liver single‐cell atlas. (H) Proportion of hepatocyte subpopulations mapped to different human hepatocyte subtypes under chow and HSHFD conditions. (I) UMAP plots showing how different mouse hepatocyte subpopulations map to human liver atlas hepatocyte subtypes. Figure S2: Enrichment of Lipid Metabolism Pathways in Different Hepatic Cell Subpopulations from the HPA Database (A) UMAP plot depicting the clustering of different hepatic cell subpopulations based on single‐cell RNA‐seq data from human liver, with a bar chart indicating the number of cells in each subpopulation. The distinct clusters represent various hepatic cell types, including hepatocytes, T‐cells, Kupffer cells, and others. (B) UMAP plot illustrating the enrichment of the fatty acid biosynthesis pathway across different hepatic cell subpopulations, highlighting specific clusters with elevated pathway activity. (C) UMAP plot showing the enrichment of the fatty acid elongation pathway within various hepatic cell subpopulations, indicating the differential involvement of this pathway among different cell types. (D) UMAP plot displaying the enrichme [file JCMM-30-e71274-s001.zip › jcmm71274-sup-0010-FigureS10@supplementaryfigure10.jpg]

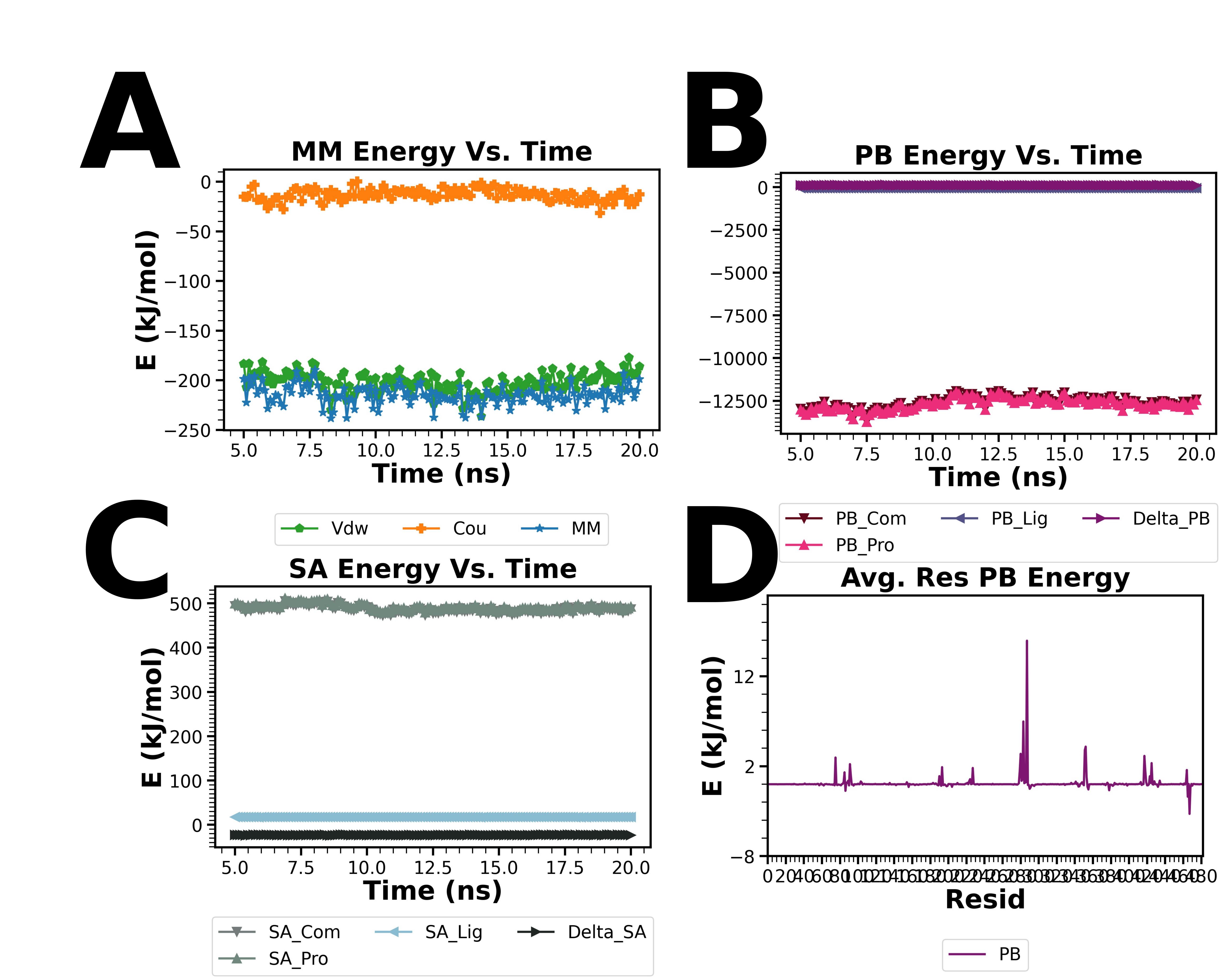

Supplement: Supplementary file 1 — Figure S1: Single‐cell analysis of hepatocyte subpopulations under chow and high‐sugar, high‐fat diet (HSHFD) conditions. (A) Metabolic activity scores across different hepatocyte subpopulations. (B) Heatmap showing activity of various metabolic pathways in different hepatocyte subpopulations. (C) UMAP plots illustrating fatty acid elongation activity in hepatocyte subpopulations under chow and HSHFD conditions. (D) UMAP plots showing fatty acid biosynthesis activity in hepatocyte subpopulations under chow and HSHFD conditions. (E) UMAP plots depicting fatty acid degradation activity in hepatocyte subpopulations under chow and HSHFD conditions. (F) UMAP plots showing activity of xenobiotic metabolism by cytochrome P450 in hepatocyte subpopulations under chow and HSHFD conditions. (G) Mapping of hepatocyte subpopulations from chow and HSHFD conditions to human liver single‐cell atlas. (H) Proportion of hepatocyte subpopulations mapped to different human hepatocyte subtypes under chow and HSHFD conditions. (I) UMAP plots showing how different mouse hepatocyte subpopulations map to human liver atlas hepatocyte subtypes. Figure S2: Enrichment of Lipid Metabolism Pathways in Different Hepatic Cell Subpopulations from the HPA Database (A) UMAP plot depicting the clustering of different hepatic cell subpopulations based on single‐cell RNA‐seq data from human liver, with a bar chart indicating the number of cells in each subpopulation. The distinct clusters represent various hepatic cell types, including hepatocytes, T‐cells, Kupffer cells, and others. (B) UMAP plot illustrating the enrichment of the fatty acid biosynthesis pathway across different hepatic cell subpopulations, highlighting specific clusters with elevated pathway activity. (C) UMAP plot showing the enrichment of the fatty acid elongation pathway within various hepatic cell subpopulations, indicating the differential involvement of this pathway among different cell types. (D) UMAP plot displaying the enrichme [file JCMM-30-e71274-s001.zip › jcmm71274-sup-0011-FigureS11@supplementaryfigure11.jpg]

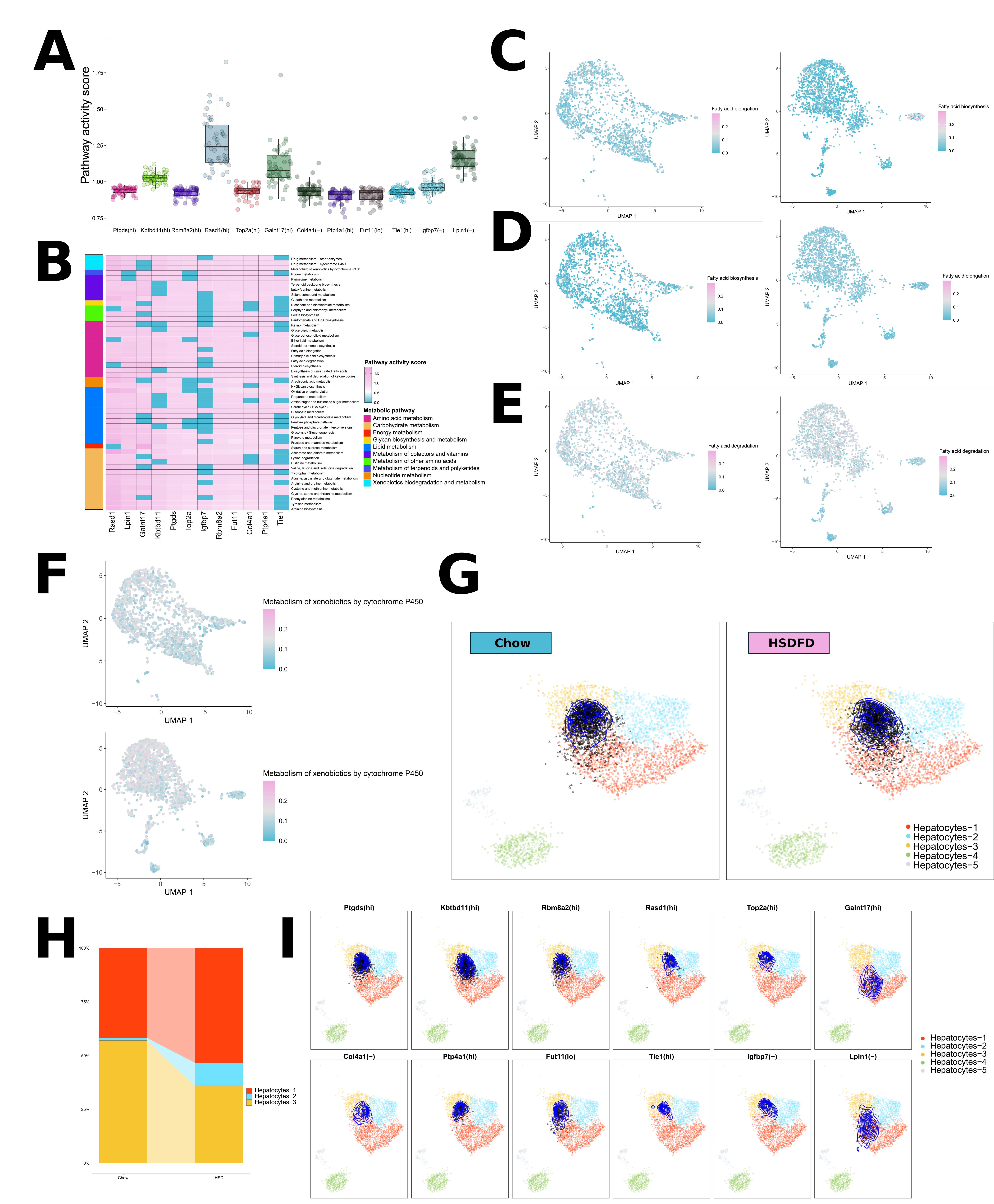

Supplement: Supplementary file 1 — Figure S1: Single‐cell analysis of hepatocyte subpopulations under chow and high‐sugar, high‐fat diet (HSHFD) conditions. (A) Metabolic activity scores across different hepatocyte subpopulations. (B) Heatmap showing activity of various metabolic pathways in different hepatocyte subpopulations. (C) UMAP plots illustrating fatty acid elongation activity in hepatocyte subpopulations under chow and HSHFD conditions. (D) UMAP plots showing fatty acid biosynthesis activity in hepatocyte subpopulations under chow and HSHFD conditions. (E) UMAP plots depicting fatty acid degradation activity in hepatocyte subpopulations under chow and HSHFD conditions. (F) UMAP plots showing activity of xenobiotic metabolism by cytochrome P450 in hepatocyte subpopulations under chow and HSHFD conditions. (G) Mapping of hepatocyte subpopulations from chow and HSHFD conditions to human liver single‐cell atlas. (H) Proportion of hepatocyte subpopulations mapped to different human hepatocyte subtypes under chow and HSHFD conditions. (I) UMAP plots showing how different mouse hepatocyte subpopulations map to human liver atlas hepatocyte subtypes. Figure S2: Enrichment of Lipid Metabolism Pathways in Different Hepatic Cell Subpopulations from the HPA Database (A) UMAP plot depicting the clustering of different hepatic cell subpopulations based on single‐cell RNA‐seq data from human liver, with a bar chart indicating the number of cells in each subpopulation. The distinct clusters represent various hepatic cell types, including hepatocytes, T‐cells, Kupffer cells, and others. (B) UMAP plot illustrating the enrichment of the fatty acid biosynthesis pathway across different hepatic cell subpopulations, highlighting specific clusters with elevated pathway activity. (C) UMAP plot showing the enrichment of the fatty acid elongation pathway within various hepatic cell subpopulations, indicating the differential involvement of this pathway among different cell types. (D) UMAP plot displaying the enrichme [file JCMM-30-e71274-s001.zip › jcmm71274-sup-0001-FigureS1@supplementaryfigure1.jpg]

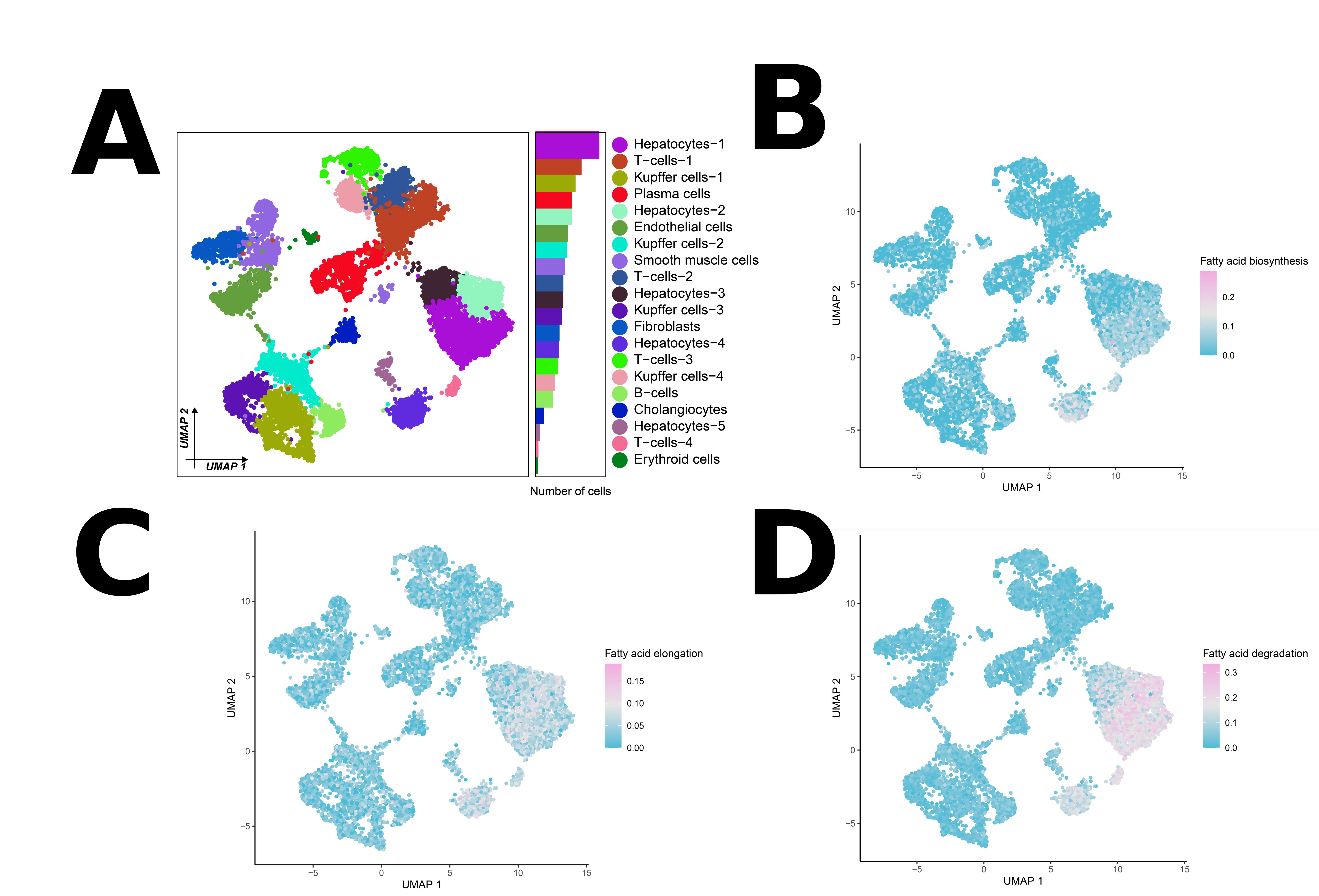

Supplement: Supplementary file 1 — Figure S1: Single‐cell analysis of hepatocyte subpopulations under chow and high‐sugar, high‐fat diet (HSHFD) conditions. (A) Metabolic activity scores across different hepatocyte subpopulations. (B) Heatmap showing activity of various metabolic pathways in different hepatocyte subpopulations. (C) UMAP plots illustrating fatty acid elongation activity in hepatocyte subpopulations under chow and HSHFD conditions. (D) UMAP plots showing fatty acid biosynthesis activity in hepatocyte subpopulations under chow and HSHFD conditions. (E) UMAP plots depicting fatty acid degradation activity in hepatocyte subpopulations under chow and HSHFD conditions. (F) UMAP plots showing activity of xenobiotic metabolism by cytochrome P450 in hepatocyte subpopulations under chow and HSHFD conditions. (G) Mapping of hepatocyte subpopulations from chow and HSHFD conditions to human liver single‐cell atlas. (H) Proportion of hepatocyte subpopulations mapped to different human hepatocyte subtypes under chow and HSHFD conditions. (I) UMAP plots showing how different mouse hepatocyte subpopulations map to human liver atlas hepatocyte subtypes. Figure S2: Enrichment of Lipid Metabolism Pathways in Different Hepatic Cell Subpopulations from the HPA Database (A) UMAP plot depicting the clustering of different hepatic cell subpopulations based on single‐cell RNA‐seq data from human liver, with a bar chart indicating the number of cells in each subpopulation. The distinct clusters represent various hepatic cell types, including hepatocytes, T‐cells, Kupffer cells, and others. (B) UMAP plot illustrating the enrichment of the fatty acid biosynthesis pathway across different hepatic cell subpopulations, highlighting specific clusters with elevated pathway activity. (C) UMAP plot showing the enrichment of the fatty acid elongation pathway within various hepatic cell subpopulations, indicating the differential involvement of this pathway among different cell types. (D) UMAP plot displaying the enrichme [file JCMM-30-e71274-s001.zip › jcmm71274-sup-0002-FigureS2@supplementaryfigure2.jpg]

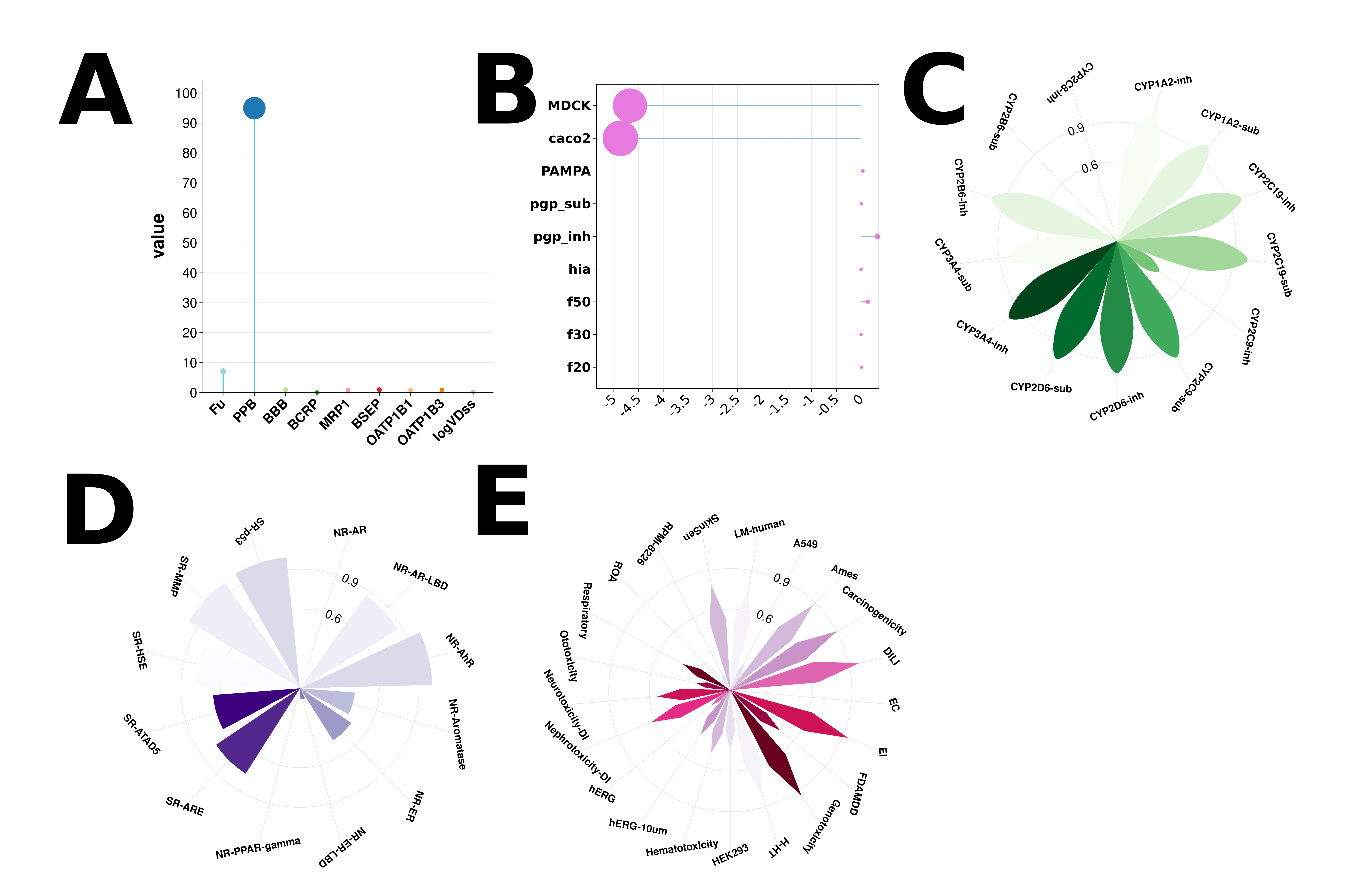

Supplement: Supplementary file 1 — Figure S1: Single‐cell analysis of hepatocyte subpopulations under chow and high‐sugar, high‐fat diet (HSHFD) conditions. (A) Metabolic activity scores across different hepatocyte subpopulations. (B) Heatmap showing activity of various metabolic pathways in different hepatocyte subpopulations. (C) UMAP plots illustrating fatty acid elongation activity in hepatocyte subpopulations under chow and HSHFD conditions. (D) UMAP plots showing fatty acid biosynthesis activity in hepatocyte subpopulations under chow and HSHFD conditions. (E) UMAP plots depicting fatty acid degradation activity in hepatocyte subpopulations under chow and HSHFD conditions. (F) UMAP plots showing activity of xenobiotic metabolism by cytochrome P450 in hepatocyte subpopulations under chow and HSHFD conditions. (G) Mapping of hepatocyte subpopulations from chow and HSHFD conditions to human liver single‐cell atlas. (H) Proportion of hepatocyte subpopulations mapped to different human hepatocyte subtypes under chow and HSHFD conditions. (I) UMAP plots showing how different mouse hepatocyte subpopulations map to human liver atlas hepatocyte subtypes. Figure S2: Enrichment of Lipid Metabolism Pathways in Different Hepatic Cell Subpopulations from the HPA Database (A) UMAP plot depicting the clustering of different hepatic cell subpopulations based on single‐cell RNA‐seq data from human liver, with a bar chart indicating the number of cells in each subpopulation. The distinct clusters represent various hepatic cell types, including hepatocytes, T‐cells, Kupffer cells, and others. (B) UMAP plot illustrating the enrichment of the fatty acid biosynthesis pathway across different hepatic cell subpopulations, highlighting specific clusters with elevated pathway activity. (C) UMAP plot showing the enrichment of the fatty acid elongation pathway within various hepatic cell subpopulations, indicating the differential involvement of this pathway among different cell types. (D) UMAP plot displaying the enrichme [file JCMM-30-e71274-s001.zip › jcmm71274-sup-0003-FigureS3@supplementaryfigure3.jpg]
